# Smoking as a mediator in the association between major depressive disorder and schizophrenia on lung cancer risk: a bidirectional/multivariable and mediation Mendelian randomization study

**DOI:** 10.3389/fpsyt.2024.1367858

**Published:** 2024-08-08

**Authors:** Xirong Zhu, Ruizhi Ye, Xianming Jiang, Jing Zhang

**Affiliations:** ^1^ Taizhou Cancer Hospital, Taizhou, China; ^2^ Taizhou Key Laboratory of Minimally Invasive Interventional Therapy & Artificial Intelligence, Taizhou, China; ^3^ Wenling Hospital of Traditional Chinese Medicine, Wenling, China; ^4^ Department of General Practice, Wenling, China

**Keywords:** major depressive disorder (MDD), schizophrenia, lung cancer, causal effect, Mendelian randomization, mediation effect

## Abstract

**Background & Aims:**

Major depressive disorder and schizophrenia have been hypothesized to be closely associated with cancer. However, the associations between these psychiatric conditions and the development of lung cancer remain uncertain. This study aimed to explore the causal relationship among major depressive disorder, schizophrenia, and the risk of lung cancer.

**Methods:**

Two-sample bidirectional/multivariable and mediation Mendelian randomization (MR) analyses were conducted. Genome-wide summary data on major depressive disorder (N=500,199) and schizophrenia (N=127,906) were utilized. Data on the risk of lung cancer (overall, adenocarcinoma, and squamous cell) were collected from a cohort of individuals of European ancestry (N=27,209). Three smoking-related behaviors (smoking initiation, pack years of smoking, and cigarettes smoked per day) were included in the multivariable and mediation MR analyses.

**Results:**

Patients with schizophrenia had a significantly greater risk of developing lung cancer (odds ratio (OR) = 1.144, 95% confidence interval (95% CI): 1.048-1.248, P = 0.003). The number of cigarettes smoked per day partially mediated the relationship between schizophrenia and the overall risk of lung cancer (OR = 1.185, 95% CI: 1.112-1.264, P = 0.021, proportion of mediation effect: 61.033%). However, there is no reliable evidence indicating an association between major depressive disorder and the risk of lung cancer (overall, adenocarcinoma, and squamous cell cancer).

**Conclusions:**

The findings indicated an association between schizophrenia and an increased risk of lung cancer, with smoking served as a partial mediator. When smoking was included in the regression analysis, the explanatory power of schizophrenia diagnosis was reduced, suggesting that smoking may be an important causal contributor to lung cancer in this population. Given the high prevalence of smoking among individuals with schizophrenia, these results underscore the need for further research to explore the underlying mechanisms of smoking’s impact. Consequently, greater emphasis should be placed on monitoring the respiratory health of individuals with schizophrenia and implementing early interventions to address smoking-related behaviors.

## Introduction

Major depressive disorder and schizophrenia are among the top ten mental disorders globally and have a significant impact on individuals’ mental and physical well-being (1, 2). By 2015, the global prevalence of depression had surpassed 300 million individuals (3). Concurrently, research has shown a 49.86% increase in the global incidence rate of depression from 1990 to 2017 (4). Schizophrenia is widely recognized as the most severe mental disorder, as it poses a significant risk to the well-being of individuals with schizophrenia and may also endanger the safety of others (2). According to available data from reputable institutions on schizophrenia, the lifetime risk of developing schizophrenia is estimated to be 1% for the general population, and both men and women are equally affected (5). Individuals with schizophrenia frequently manifest prominent psychiatric symptoms, including delusions, hallucinations, and disordered thinking, which adversely impact their overall quality of life (6). Numerous risk factors are recognized to be closely associated with major depressive disorder and schizophrenia, but due to their complex aetiology, additional research is warranted (1, 2). Recently, many studies have shown a connection between smoking-related behaviors and severe depression and schizophrenia, which significantly contribute to the onset and progression of major depressive disorder and schizophrenia (7).

Lung cancer is a prevalent malignancy and is responsible for the greatest number of cancer-related deaths globally, accounting for approximately 2 million newly diagnosed cases and 1.76 million deaths annually (8). In 2018, the United States reported over 230,000 incident cases of lung cancer, surpassing the combined mortality of breast, prostate, and colon cancer (9). Age is a significant risk factor for lung cancer, with a greater incidence observed in males than in females (10). Furthermore, chronic lung diseases, genetic factors, air pollution, kitchen fumes, long-term smoking, and second-hand smoke are all risk factors for lung cancer, among which long-term smoking is considered an independent and important risk factor for lung cancer (11–13). Several studies have indicated a greater prevalence of smoking and increased vulnerability to nicotine addiction among individuals with severe depression and schizophrenia, which may contribute to a heightened risk of developing lung cancer. Concurrently, studies conducted by Benchalak Maneeton et al. revealed a heightened prevalence of major depressive disorder in individuals suffering from lung cancer, suggesting a potential correlation between major depressive disorder and lung cancer (14). Furthermore, research by Merete Nordentoft et al. has shown that patients with schizophrenia face an increased risk of mortality from various cancers, notably lung cancer (15). However, substantial evidence supporting the association between major depressive disorder, schizophrenia, and the risk of lung cancer remains limited (16–18). These findings accentuate the urgent need to delve into the causal connections between major depression, schizophrenia, and the elevated risk of lung cancer development.

In recent years, with the rapid development of technology, an increasing number of Genome-Wide Association Study (GWAS) databases have been utilized for scientific research (19). Moreover, Mendelian randomization (MR), a causal inference method, has wider application prospects (20). Owing to the principle of allele distribution in genetic variation, MR analysis can effectively mitigate potential confounding factors, and is often referred to as a “natural randomized controlled trial” (21). Single nucleotide polymorphisms (SNPs) strongly correlated with exposure factors can be employed as instrumental variables (IVs) to elucidate the potential causal relationship between exposure and outcomes (22). Additionally, two-sample bidirectional and multivariable MR analyses, which can serve as effective supplements to MR research, are wildly utilized to uncover certain genetic associations between exposures and outcomes (23).

To our knowledge, there is limited research using MR to investigate the causal effects of major depressive disorder and schizophrenia on the risk of lung cancer (overall, adenocarcinoma, and squamous cell). Consequently, our aim was to discover these causal relationships by utilizing two-sample bidirectional and multivariable MR analyses. Furthermore, we investigated the mediating effects of three smoking-related behaviors (smoking initiation, pack years of smoking, and cigarettes smoked per day) on the relationship between major depressive disorder and schizophrenia and the risk of lung cancer (overall, adenocarcinoma, and squamous cell).

## Materials and methods

### Mendelian randomization study design

**Figure 1:**
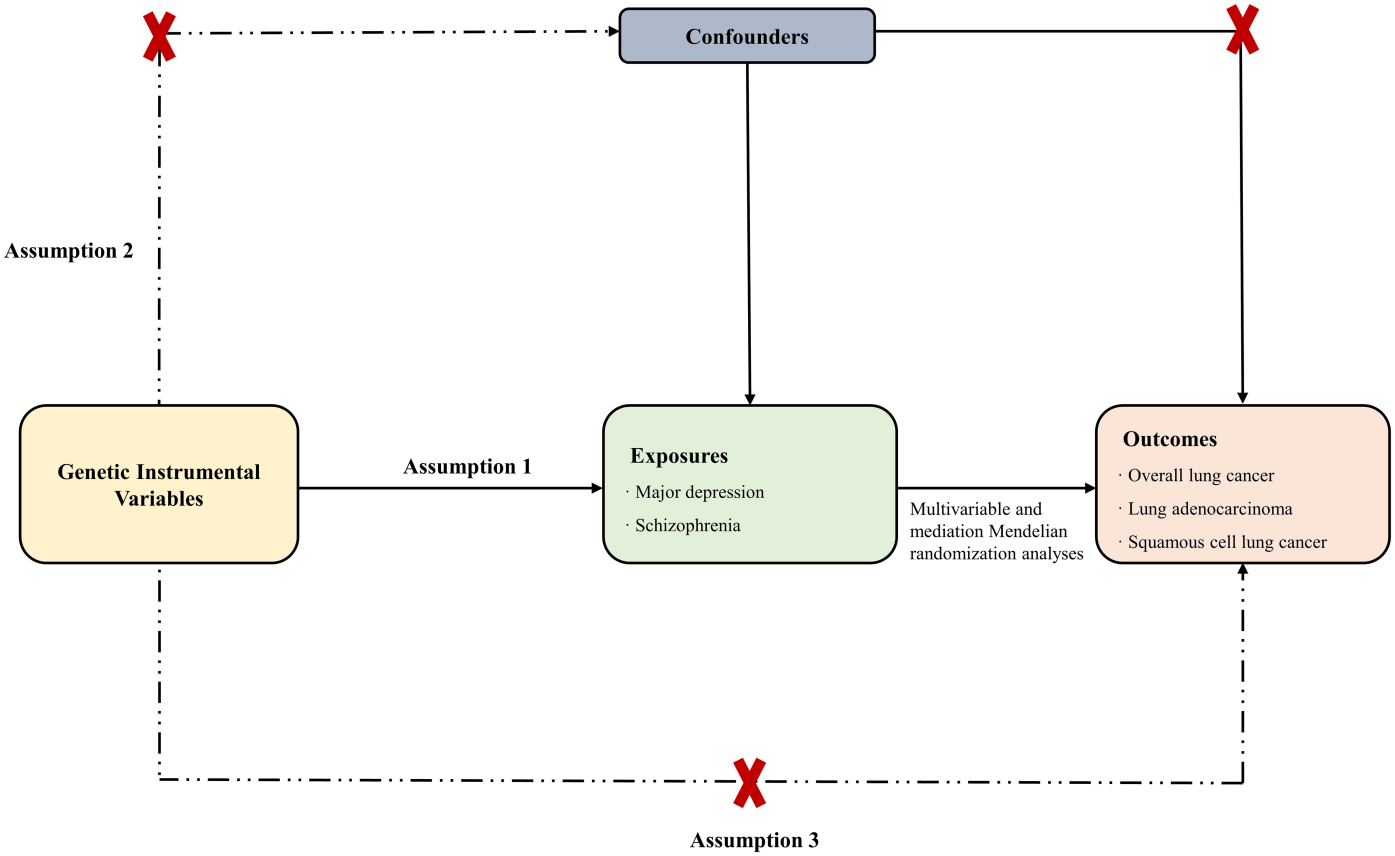
Directed acyclic graph of the Mendelian randomization study.

**Figure 2:**
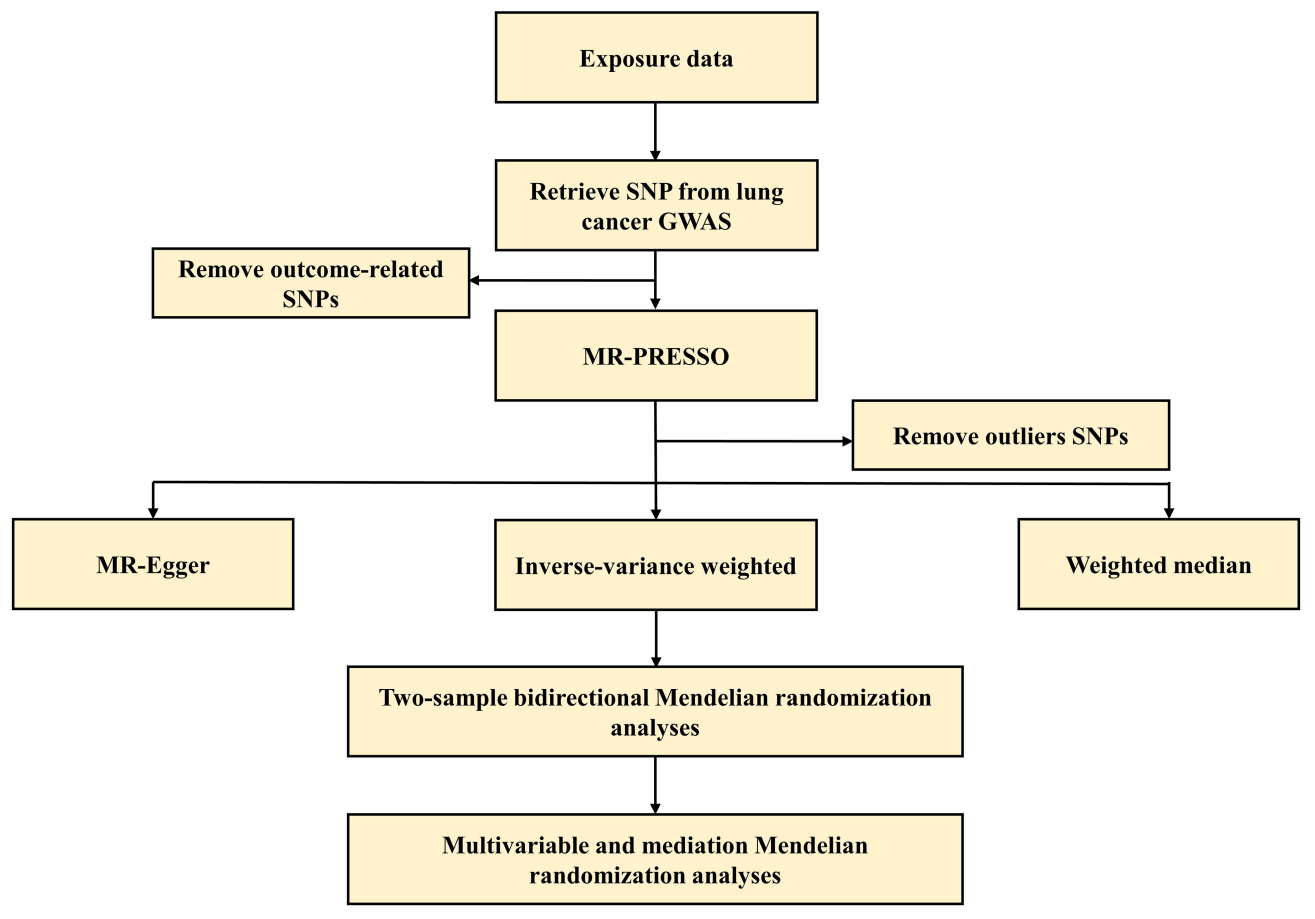
The flowchart of this Mendelian randomization study. GWAS, genome-wide association studies; MR-PRESSO, MR Pleiotropy RESidual Sum and Outlier; SNP, single nucleotide polymorphisms.

The Mendelian randomization study was conducted according to the STROBE-MR statement (24). Furthermore, two-sample bidirectional/multivariable Mendelian randomization and mediation analysis were employed in this study. To ensure the reliability of the study, three assumptions were made: (i) the selected IVs are strongly associated with major depressive disorder and schizophrenia, (ii) the IVs are not connected with other confounding factors, and (iii) the IVs only affect the outcome through the exposure (**Figure 1**) (23). Causal relationships were evaluated using the IVW method, while additional methods such as weighted median, MR-Egger, and MR-PRESSO were employed to ensure the reliability of the findings. Additionally, bidirectional MR analysis revealed a causal relationship between exposures and outcomes. Since smoking-related behaviors are important risk factors for lung cancer, multivariate MR analysis included these confounding factors. Mediation analysis was further conducted to examine the mediating role of smoking-related behaviors between exposures and outcomes. The flowchart of the study is shown in **Figure 2**.

### Exposure and outcome data sources

Data on major depressive disorder and schizophrenia were collected from two large European ancestry cohorts. This study utilized GWAS summary data from the PGC, consisting of 170,756 cases and 329,443 controls of European ancestry, to examine major depressive disorder (25). Severe depression patients in the study were defined as those diagnosed with severe depression during hospitalization or based on medical health records, or those who self-reported depression or had received relevant treatment (25). The GWAS summary data on schizophrenia were derived from the PGC consortium, which included 52,017 cases and 75,889 controls of European ancestry (26). Clinical experts diagnosed most of the patients, including individuals with schizophrenia and schizoaffective disorder, while others were diagnosed based on research-based assessments (26). Smoking-related behaviors, including smoking initiation, pack years of smoking, and cigarettes smoked per day, were considered as mediating factors. Recent WAS of smoking initiation and cigarettes smoked per day from the GSCAN consortium identified 607,291 and 249,752 individuals of European ancestry, respectively (27). In addition, a cohort of 142,387 individuals of European ancestry was used to summarize the number of pack years of smoking. These smoking-related behaviors were assessed through self-report questionnaires and initial assessments (27). Additionally, the outcome data on overall lung cancer, lung adenocarcinoma, and squamous cell lung cancer were sourced from a European ancestry cohort in the International Lung Cancer Consortium (ILCCO), which included 11,348, 3,442, and 3,275 patients, respectively (28). The outcome data were collected from a non-overlapping population to effectively minimize bias (28). Detailed information on all exposure and outcome data is provided in **Supplementary Table S1**.

Furthermore, recognizing the impact of the sample overlap rate between the exposure and outcome databases on the study’s results, we meticulously quantified the relevant sample overlap rate. Simultaneously, utilizing the correct methodologies (https://sb452.shinyapps.io/overlap/), we assessed the statistical bias and the likelihood of type I error resulting from the sample overlap rate (29). The results revealed that the sample overlap rate in our study was less than 10% (**Supplementary Table S2**), imposing a negligible influence on the research outcomes (30).

### Selected IVs

The IVs were carefully selected to ensure a strong connection between major depressive disorder and schizophrenia. IVs with a P-value < 5×10^-8^ were considered significant and selected. Moreover, IVs with a low likelihood of linkage disequilibrium (LD) (R^2^ < 0.001) and a substantial physical distance (≥10,000 KB) were selected (31). A higher F-value (F > 10) was used to mitigate potential biases caused by weak instruments in this study (32). Furthermore, IVs selected for participation in this study were scanned utilizing the PhenoScanner tool (http://www.phenoscanner.medschl.cam.ac.uk/) to exclude the potential confounding factors (33); if any SNP was connected with the outcomes and confounding factors, they were removed (**Supplementary Table S3**). **Supplementary Table S4** presents a comprehensive list of all the instrumental variables utilized in this study.

### Statistical analysis

All analyses were performed using R software (*Version 4.2.1*). The analyses utilized the “TwoSampleMR” (*Version 0.5.6*), and “MR-PRESSO” (*Version 1.0*) R packages. All statistical analyses were conducted using a two-sided approach. To mitigate bias, a Bonferroni-corrected significance level of P-value < 0.0083 (0.05/6) was used (34). P-values between 0.0083 and 0.05 were considered suggestive of a potential association. P-values greater than 0.05 indicated the absence of a statistically significant association between the exposures and outcomes.

## Results

### Two-sample bidirectional MR study

**Figure 3:**
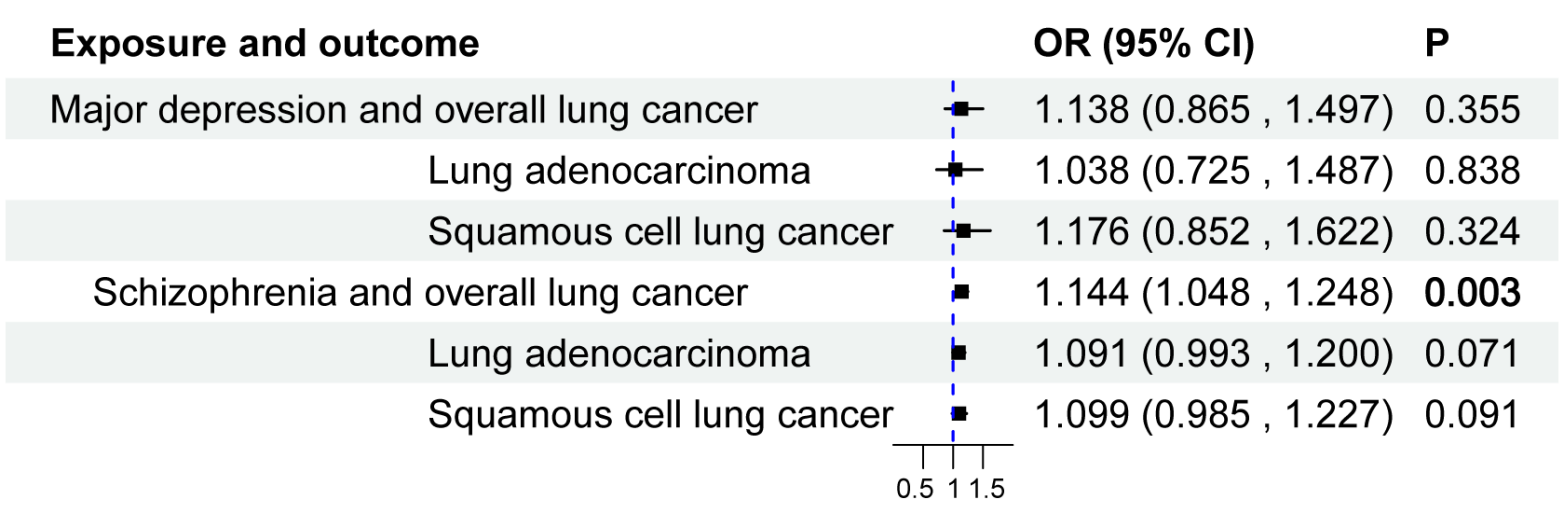
Causal effect of major depressive disorder and schizophrenia on the risk of Lung Cancer. 95%CI, 95% confidence interval; OR, odds ratio.

The IVW results for major depressive disorder and schizophrenia’s impact on the overall risk of lung cancer, lung adenocarcinoma, and squamous cell lung cancer are presented in **Table 1**, **Figure 3**. Schizophrenia was found to have a genetic connection with an increased risk of overall lung cancer (OR = 1.144, 95% CI: 1.048-1.248, P = 0.003). However, no significant associations were observed between schizophrenia and the risk of lung adenocarcinoma or squamous cell lung cancer. Similarly, there was no noteworthy association between major depressive disorder and the risk of lung cancer, lung adenocarcinoma, or squamous cell lung cancer. To mitigate potential reverse causal effects, we conducted a reverse MR analysis to evaluate the impact of the overall risk of lung cancer, lung adenocarcinoma, and squamous cell lung cancer on major depressive disorder and schizophrenia. The IVW results of the reverse MR analysis indicated no reverse causal effects of the risk of overall lung cancer, lung adenocarcinoma, or squamous cell lung cancer on major depressive disorder and schizophrenia. The results of the reverse MR analysis can be found in **Supplementary Table S5**.

**Table 1:** Causal effect of major depressive disorder and schizophrenia on Lung Cancer.

Exposure	Outcome	Method	Number of IVs	OR (95% CI)	P-Value
Major depressive disorder	Overall lung cancer	IVW	47	1.138 (0.865, 1.497)	0.355
		WM	47	1.122 (0.837, 1.504)	0.443
		MR Egger	47	0.266 (0.059, 1.194)	0.091
	Lung adenocarcinoma	IVW	47	1.038 (0.725, 1.487)	0.838
		WM	47	0.825 (0.531, 1.281)	0.391
		MR Egger	47	0.686 (0.092, 5.116)	0.715
	Squamous cell lung cancer	IVW	47	1.176 (0.852, 1.622)	0.324
		WM	47	1.046 (0.679, 1.613)	0.838
		MR Egger	47	0.315 (0.052, 1.893)	0.213
Schizophrenia	Overall lung cancer	IVW	143	1.144 (1.048, 1.248)	0.003*
		WM	143	1.103 (1.019, 1.194)	0.015*
		MR Egger	143	1.187 (0.833, 1.692)	0.345
	Lung adenocarcinoma	IVW	143	1.091 (0.993, 1.200)	0.071
		WM	143	1.046 (0.934, 1.171)	0.434
		MR Egger	143	1.165 (0.796, 1.705)	0.434
	Squamous cell lung cancer	IVW	143	1.099 (0.985, 1.227)	0.091
		WM	143	1.019 (0.905, 1.147)	0.758
		MR Egger	143	1.204 (0.767, 1.889)	0.421

95% CI, 95% confidence interval; IV, instrumental variables; IVW, inverse-variance weighted; OR, odds ratio; WM, weighted median; *statistically significant results (P-Value < 0.05).

Values in bold indicate statistical significance.

### Multivariable and mediation MR study

The multivariable MR analyses considered three smoking-related behaviors: smoking initiation, pack years of smoking, and cigarettes smoked per day. After adjusting for these smoking-related behaviors, we did not find any evidence of a causal relationship between major depressive disorder, schizophrenia, and overall risk of lung cancer, lung adenocarcinoma, or squamous cell lung cancer (**Supplementary Table S6**). These findings suggested that the observed effects of major depressive disorder and schizophrenia on the risk of these lung cancers are potentially mediated by smoking-related behaviors. Additionally, the mediation analysis demonstrated that cigarettes smoked per day mediated the association between schizophrenia and the overall risk of lung cancer with an OR of 1.185 (95% CI: 1.112-1.264, P = 0.021), and a mediation effect of 61.033%. The mediation effect is presented in **Table 2**.

**Table 2:** Mediation effect of smoking-related behaviors on schizophrenia and Lung Cancer.

Mediation factors	Association between schizophrenia and mediation factors		Association between mediation factors and lung cancer		Mediation effect of smoking-related behaviors on schizophrenia and lung cancer		
	OR (95% CI)	P-Value	OR (95% CI)	P-Value	OR (95% CI)	Proportion of mediation effect	P-Value
**Smoking initiation**	1.045 (1.022, 1.069)	1.389E-04*	1.648 (1.372, 1.979)	9.058E-08*	1.022 (0.933, 1.120)	16.390%	0.638
**Pack years of smoking**	1.019 (1.001, 1.036)	0.035*	2.116 (1.428, 3.198)	9.370E-04*	1.041 (0.063, 17.224)	30.084%	0.978
**Cigarettes smoked per day**	1.046 (1.008, 1.086)	0.018*	2.631 (1.883, 3.675)	1.411E-08*	1.185 (1.112, 1.264)	61.033%	0.021*

95% CI, 95% confidence interval; OR, odds ratio; *statistically significant results (P-Value < 0.05).

Values in bold indicate statistical significance.

### Sensitivity analysis

The F-values of all IVs were greater than 10, indicating their ability to mitigate potential biases (**Supplementary Table S4**). To assess the validity of the results obtained from this MR analysis, we conducted comprehensive sensitivity analyses, which included the heterogeneity test, pleiotropy test, and MR-PRESSO test. Theresults of these analyses revealed no horizontal pleiotropy or heterogeneity (**Supplementary Tables S7**, **S8**). Moreover, the leave-one-out plots demonstrated the causal effects of schizophrenia on the overall risk of lung cancer (**Supplementary Figure S1**). Additionally, both the scatter plot and funnel plot for schizophrenia and the overall risk of lung cancer indicated the reliability of the MR results (**Supplementary Figures S1**, **S2**).

## Discussion

In this two-sample bidirectional/multivariable and mediation MR study, we examined the causal relationships between major depressive disorder, schizophrenia, and the overall risk of lung cancer, lung adenocarcinoma, and squamous cell lung cancer. Based on two-sample bidirectional MR analyses, schizophrenia was found to be significantly associated with an increased overall risk of lung cancer. Moreover, we identified a partial mediation effect of cigarettes smoked per day on the association between schizophrenia and the overall risk of lung cancer, providing further insight into the impact of schizophrenia on overall lung cancer development.

Major depressive disorder and schizophrenia, two highly debilitating mental illnesses, greatly affect human health (35, 36). Numerous studies have demonstrated the detrimental effects of depression and schizophrenia on the incidence, progression, and prognosis of cancer (15, 37). Studies have suggested a potential stress pathway shared between depression and cancer, wherein certain pro-inflammatory mediators impede the regulatory feedback of the hypothalamic-pituitary-adrenal axis, which is mediated by glucocorticoids (38). Additionally, several MR studies have identified correlations between depression and the incidence and progression of prostate and breast cancer (18, 39). Nevertheless, our study did not uncover a causal link between severe depression and the risk of lung cancer (including overall, adenocarcinoma, and squamous cell types). Moreover, even after accounting for three smoking-related behaviors in the multivariate MR analysis, we observed no causal connection between depression and lung cancer risk. A 21-year longitudinal investigation revealed that major depressive disorder is linked to an increased frequency of daily smoking and higher rates of nicotine dependence; however, the causal relationship between smoking and depression remains elusive (40). Concurrently, Patrícia Pelufo Silveira et al. identified 11 gene loci in women and a single gene locus in men that were significantly associated with the major depressive disorder phenotype through gender-specific GWAS analysis of UK Biobank data. Given the notably greater incidence of lung cancer in men than in women, our inability to establish a link between major depression and lung cancer risk may stem from gender disparities. The current limitations of GWAS databases preclude further sex-specific analysis, underscoring the imperative for future sex-stratified investigations. This suggests that depression may not be significantly linked to the development of lung cancer, emphasizing the need for further research on this relationship.

A cohort study revealed a correlation between schizophrenia and the incidence of breast cancer, possibly attributed to hyperprolactinemia resulting from patients’ long-term use of antipsychotic drugs, ultimately leading to breast cancer (41). Furthermore, certain well-established antipsychotic medications, such as phenothiazines and reserpine, have exhibited anticancer effects. This finding suggests possible shared mechanisms and pathways between schizophrenia and the onset and progression of cancer (42). In a meta-analysis of 13 cohort studies with 218,076 male participants, Fan et al. reported a correlation between schizophrenia and the likelihood of developing prostate cancer (43). By utilizing data from the Swedish cohort and genome-wide association study (GWAS) data from the International Union, a study revealed a genetic association between schizophrenia and breast cancer, identifying the shared locus 19p13 (GATAD2A), and these findings indicate a genetic overlap between the two phenotypes (44). Collectively, these studies demonstrate a strong relationship between schizophrenia and the incidence and progression of cancer, emphasizing the need to examine the underlying mechanisms involved. Our two-sample bidirectional MR analysis provided robust evidence indicating an increased risk of overall lung cancer among individuals with schizophrenia. Given the greater propensity of individuals with schizophrenia to engage in smoking, which is a significant risk factor for lung cancer, we conducted additional multivariate and mediation Mendelian randomization analyses to investigate the potential impact of smoking-related behaviors on the relationship between these two conditions. After accounting for smoking behavior in the multivariate Mendelian randomization analysis, the previously observed association between schizophrenia and the risk of lung cancer lost significance, suggesting that smoking-related behavior mediated this relationship. The mediation analysis results revealed that the number of cigarettes smoked per day partially mediates the association between schizophrenia and the overall risk of developing lung cancer. These findings clarify the link between schizophrenia and the development of lung cancer while also offering novel perspectives for future mechanism-based research.

There are several strengths in this MR study. First, the GWAS summary data for major depressive disorder, schizophrenia, and the overall risk of lung cancer, lung adenocarcinoma, and squamous cell lung cancer were collected from the European population, minimizing potential biases (30). Second, the Bonferroni-corrected analysis was utilized to reduce the potential risk of type I error (34). Third, this MR study revealed for the first time a causal relationship between major depressive disorder, schizophrenia, and overall risk of lung cancer, lung adenocarcinoma, and squamous cell lung cancer.

Several limitations exist in this MR study. First, the causal connection between schizophrenia and the overall risk of lung cancer in populations of different races remains unknown due to the inclusion of predominantly European cohorts in this MR study. Second, the genetic association between schizophrenia and the overall risk of lung cancer found in this MR study was not verified by other databases due to certain limitations. Third, future studies should extensively investigate the potential mechanisms that elucidate the connection between smoking-related behaviors, schizophrenia, and overallrisk of lung cancer.

In conclusion, our bidirectional/multivariable and mediation MR study revealed a causal relationship between schizophrenia and the overall risk of lung cancer, with smoking serving as a significant mediator. Including smoking in the analysis reduced the explanatory power of schizophrenia for lung cancer risk, suggesting that smoking is a crucial causal factor in this relationship. This finding demonstrates a heightened risk of developing lung cancer among individuals with schizophrenia, highlighting the need to prioritize lung health in these patients. Therefore, early interventions to address smoking-related behaviors are essential to mitigate the increased risk of lung cancer in this population.

## Data availability statement

The datasets presented in this study can be found in online repositories. The names of the repository/repositories and accession number(s) can be found in the article/**Supplementary Material**.

## Author contributions

XZ: Conceptualization, Data curation, Formal analysis, Writing – original draft, Writing – review & editing. RY: Data curation, Writing – review & editing. XJ: Data curation, Writing – review & editing. JZ: Conceptualization, Formal analysis, Funding acquisition, Writing – original draft, Writing – review & editing.
