# Supplementary figures and images for: Smoking as a mediator in the association between major depressive disorder and schizophrenia on lung cancer risk: a bidirectional/multivariable and mediation Mendelian randomization study
Source: Front Psychiatry. 2024 Aug 8;15:1367858. doi: 10.3389/fpsyt.2024.1367858 (PMC11338888; doi:10.3389/fpsyt.2024.1367858)

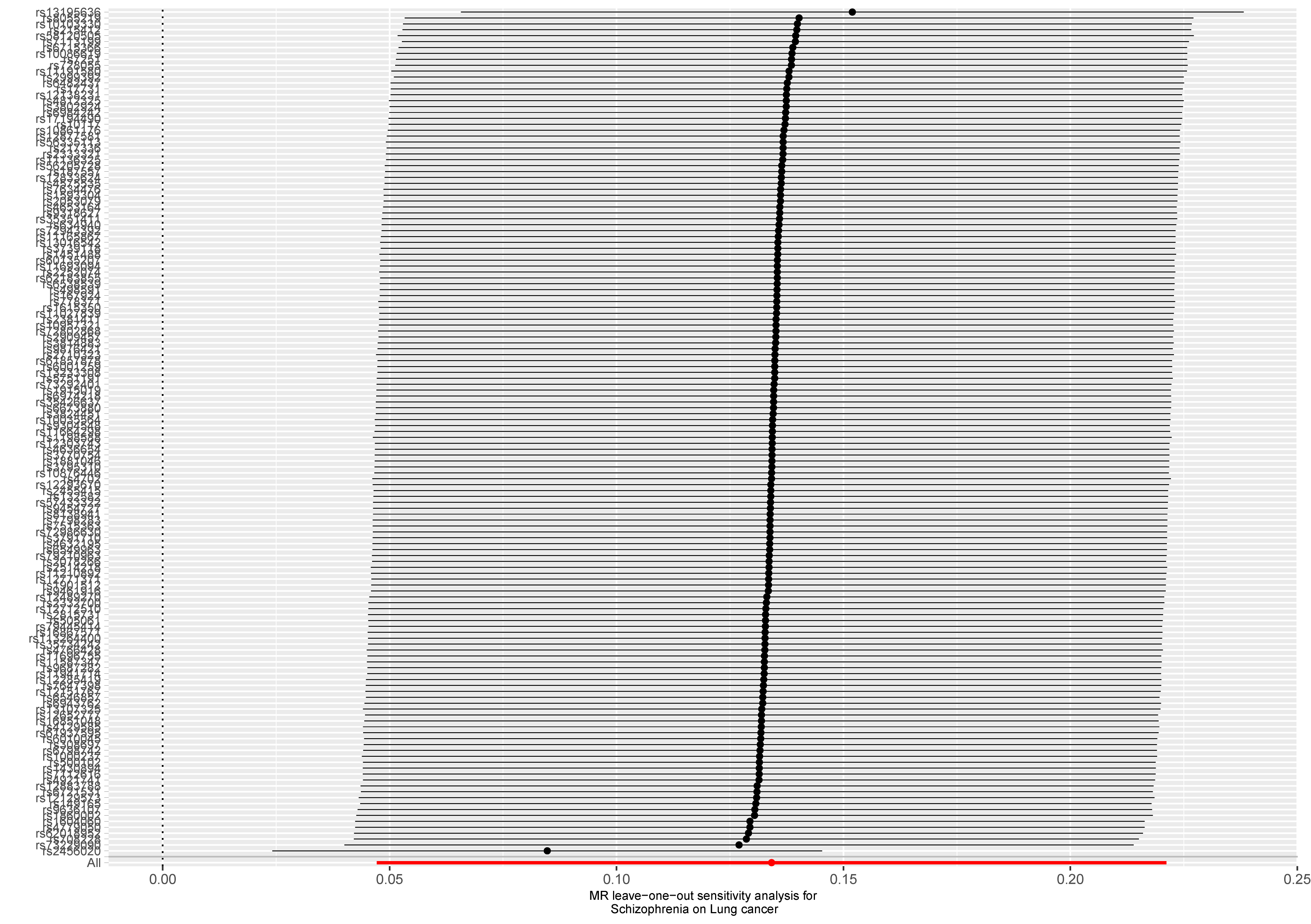

Supplement: Supplementary file 1 [file Image_1.tif]

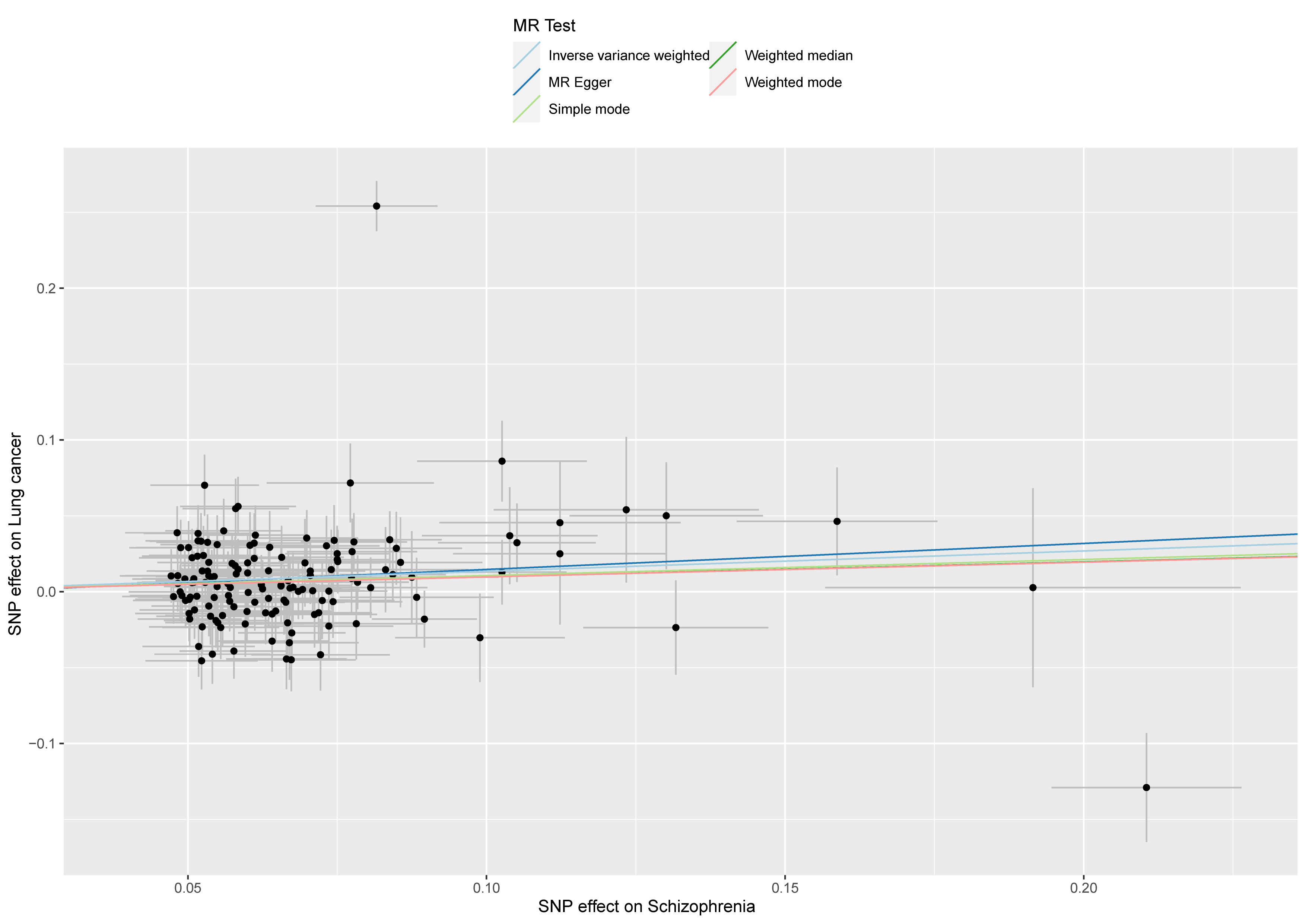

Supplement: Supplementary file 2 [file Image_2.tif]

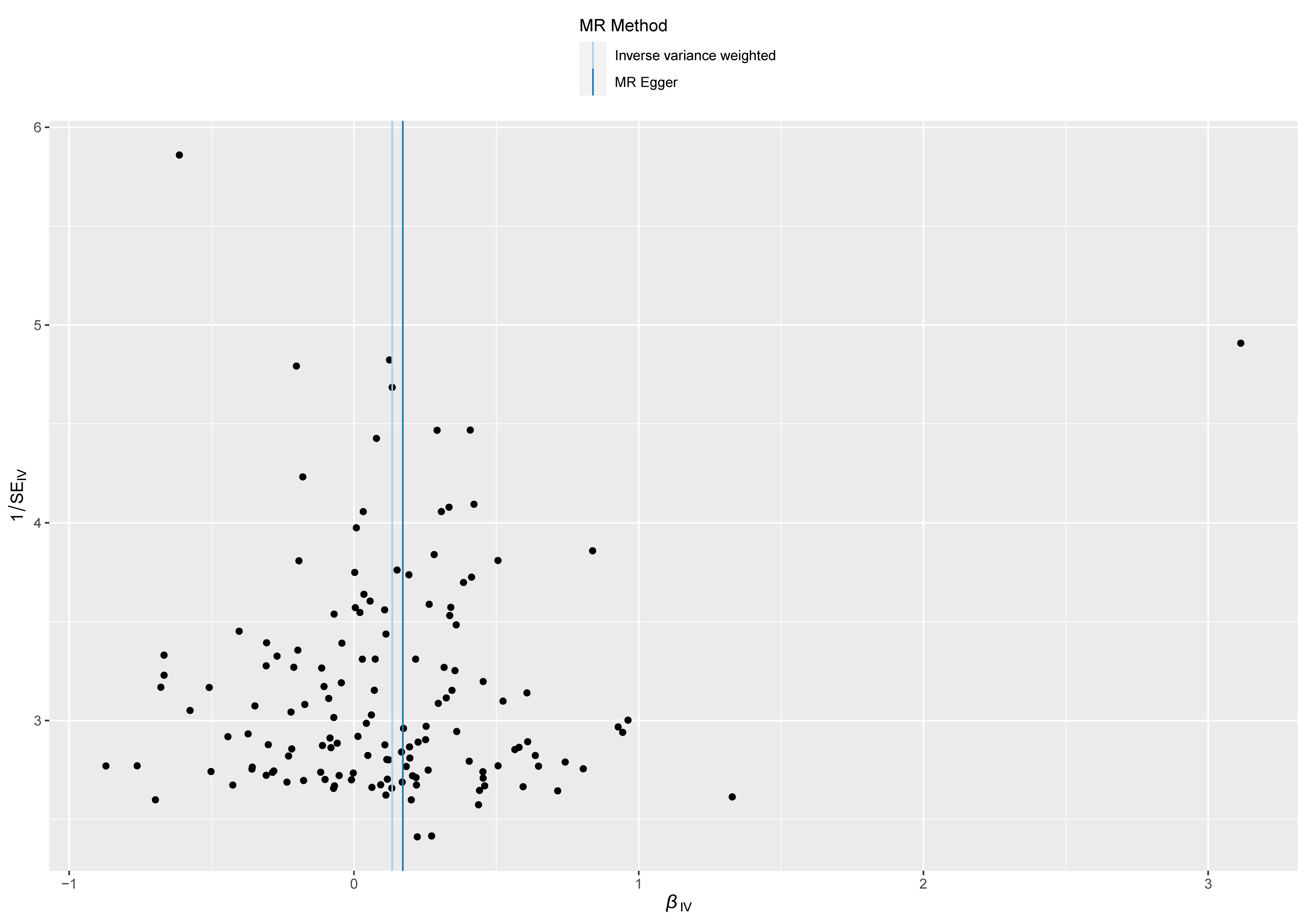

Supplement: Supplementary file 3 [file Image_3.tif]
