# Supplementary material for: Smoking as a mediator in the association between major depressive disorder and schizophrenia on lung cancer risk: a bidirectional/multivariable and mediation Mendelian randomization study
Source: Front Psychiatry. 2024 Aug 8;15:1367858. doi: 10.3389/fpsyt.2024.1367858 (PMC11338888; doi:10.3389/fpsyt.2024.1367858)
Supplement: Supplementary file 4 [file Table_1.docx]

**List of Supporting Information:**

**Supplementary Tables**

**Supplementary Table S1** | Details of GWAS summary data. **Abbreviations:** PMID: Pubmed ID; SNP: single nucleotide polymorphisms.

**Supplementary Table S2** | Sample overlap between the exposure databases and outcome databases.

**Supplementary Table S3** | Details of phenotypes related to instrumental variables found on the Phenoscanner website.

**Supplementary Table S4** | Characteristics of SNPs extracted from exposure data. **Abbreviations:** Chr: chromosome; EA: effect Allele; EAF: effect allele frequency; OA: other Allele; SE: standard error.

**Supplementary Table S5** | Causal effect of Lung Cancer on major depressive disorder and schizophrenia. **Abbreviations:** 95% CI: 95% confidence interval; IV: instrumental variables; IVW: inverse-variance weighted; OR: odds ratio; WM: weighted median.

**Supplementary Table S6** | Effects of major depressive disorder and schizophreniaon on lung cancer after regulating smoking-related behaviors by multivariate Mendelian randomization analysis. **Abbreviations:** 95% CI: 95% confidence interval; BMI: body mass index; IV: instrumental variables; IVW: inverse-variance weighted; OR: odds ratio.

**Supplementary Table S7** | Sensitivity analysis on the association between major depressive disorder, schizophrenia and Lung Cancer. **Abbreviations:** MR-PRESSO: MR Pleiotropy RESidual Sum and Outlier.

**Supplementary Table S8** | Sensitivity analysis on the association between Lung Cancer and Major depressive disorder/schizophrenia. **Abbreviations:** MR-PRESSO: MR Pleiotropy RESidual Sum and Outlier.

**Supplementary Figure Legends**

**Supplementary Figure S1** | Leave-one-out sensitivity analysis for schizophrenia on Lung Cancer.

**Supplementary Figure S2** | Scatter plot for schizophrenia on Lung Cancer.

**Supplementary Figure S3** | Funnel plot for schizophrenia on Lung Cancer.

**Supplementary Table S1** | Details of GWAS summary data.

| **Exposure / Outcome** | **Ethnicity** | **Total** | **PMID / URL** | **Author** |
| --- | --- | --- | --- | --- |
|  |  | **population** |  |  |
| **Exposure** | | | | |
| Major depressive disorder | European | 500,199 | 30718901 | Howard DM et al. |
| Schizophrenia | European | 127,906 | 35396580 | Trubetskoy V et al. |
| Smoking initiation | European | 607,291 | 30643251 | Liu M et al. |
| Pack years of smoking | European | 142,387 | <https://gwas.mrcieu.ac.uk/> | Ben Elsworth et al. |
| Cigarettes smoked per day | European | 249,752 | 30643251 | Liu M et al. |
| **Outcome** | | | | |
| Overall lung cancer | European | 27,209 | 24880342 | Wang Y et al. |
| Lung adenocarcinoma | European | 18,336 | 24880342 | Wang Y et al. |
| Squamous cell lung cancer | European | 18,313 | 24880342 | Wang Y et al. |

**Abbreviations:** PMID: Pubmed ID; SNP: single nucleotide polymorphisms.

**Supplementary Table S2 |** Sample overlap between the exposure databases and outcome databases.

| **Exposure** | **Overall lung cancer** | | | **Lung adenocarcinoma** | | | **Squamous cell lung cancer** | | |
| --- | --- | --- | --- | --- | --- | --- | --- | --- | --- |
|  | **Overlapping percentage (%)** | **Bias** | **Type I error rate** | **Overlapping percentage (%)** | **Bias** | **Type I error rate** | **Overlapping percentage (%)** | **Bias** | **Type I error rate** |
| Major depressive disorder | 3.26% | < 0.005 | 0.05 | 1.12% | < 0.005 | 0.05 | 0.78% | < 0.005 | 0.05 |
| Schizophrenia | 5.77% | < 0.005 | 0.05 | 2.57% | < 0.005 | 0.05 | 4.16% | < 0.005 | 0.05 |
| Smoking initiation | 4.58% | < 0.005 | 0.05 | 5.16% | < 0.005 | 0.05 | 4.08% | < 0.005 | 0.05 |
| Pack years of smoking | 6.73% | < 0.005 | 0.05 | 7.23% | < 0.005 | 0.05 | 2.35% | < 0.005 | 0.05 |
| Cigarettes smoked per day | 3.69% | < 0.005 | 0.05 | 3.32% | < 0.005 | 0.05 | 7.63% | < 0.005 | 0.05 |

**Supplementary Table S3 |** Details of phenotypes related to instrumental variables found on the Phenoscanner website.

| Exposure | **SNP** | **Trait** |
| --- | --- | --- |
| Major depressive disorder | rs7551758 | Major depressive disorder; Body mass index |
| Major depressive disorder | rs2568958 | Major depressive disorder; Body mass index |
| Major depressive disorder | rs10913112 | Major depressive disorder |
| Major depressive disorder | rs17641524 | Major depressive disorder |
| Major depressive disorder | rs354155 | Major depressive disorder; Body mass index |
| Major depressive disorder | rs7538938 | Major depressive disorder; Height |
| Major depressive disorder | rs4141983 | Major depressive disorder; Alanine aminotransferase levels |
| Major depressive disorder | rs2111592 | Major depressive disorder; Neuroticism conditioned on educational attainment (multi-trait conditioning and joint analysis) |
| Major depressive disorder | rs72948506 | Major depressive disorder; Household income |
| Major depressive disorder | rs35469634 | Major depressive disorder; Adult body size; Body mass index |
| Major depressive disorder | rs843812 | Major depressive disorder; Body surface area; Hip circumference |
| Major depressive disorder | rs9831648 | Major depressive disorder; Educational attainment (years of education); C-reactive protein levels |
| Major depressive disorder | rs66511648 | Major depressive disorder; Alcohol consumption (drinks per week) (MTAG); Educational attainment |
| Major depressive disorder | rs76954012 | Major depressive disorder; Insomnia |
| Major depressive disorder | rs30266 | Major depressive disorder; HDL cholesterol levels; Predicted visceral adipose tissue |
| Major depressive disorder | rs247910 | Major depressive disorder; Educational attainment (years of education); Household income (MTAG) |
| Major depressive disorder | rs7725715 | Major depressive disorder; Neuroticism; Haemorrhoidal disease |
| Major depressive disorder | rs150186873 | Major depressive disorder; Neuroticism; Well-being spectrum (multivariate analysis) |
| Major depressive disorder | rs2232423 | Major depressive disorder; Triglycerides; Cholesteryl esters to total lipids ratio in small VLDL |
| Major depressive disorder | rs9364755 | Major depressive disorder; Neonatal abstinence syndrome; Tuberculosis |
| Major depressive disorder | rs2214123 | Major depressive disorder; Blood trace element (Cu levels); Help-seeking from a GP |
| Major depressive disorder | rs2876520 | Major depressive disorder; Lobe attachment; Hip circumference adjusted for BMI |
| Major depressive disorder | rs2522831 | Major depressive disorder; Bipolar disorder (MTAG); Insomnia |
| Major depressive disorder | rs4730387 | Major depressive disorder; Body mass index; Insomnia |
| Major depressive disorder | rs150346963 | Major depressive disorder; Cortical surface area (MOSTest) |
| Major depressive disorder | rs3807865 | Major depressive disorder; General cognitive ability; HDL cholesterol levels |
| Major depressive disorder | rs10235664 | Major depressive disorder; Educational attainment; Feeling miserable |
| Major depressive disorder | rs59082935 | Major depressive disorder |
| Major depressive disorder | rs62535714 | Major depressive disorder; Sleep duration; Suicidality |
| Major depressive disorder | rs1931388 | Major depressive disorder; Neuroticism; Positive affect; Well-being spectrum (multivariate analysis) |
| Major depressive disorder | rs59283172 | Major depressive disorder; General cognitive ability; Educational attainment |
| Major depressive disorder | rs2418449 | Major depressive disorder; Caudal middle frontal gyrus volume; Educational attainment |
| Major depressive disorder | rs1021363 | Major depressive disorder; General risk tolerance (MTAG); Irritable bowel syndrome (MTAG) |
| Major depressive disorder | rs198457 | Major depressive disorder; Serum metabolite ratios in chronic kidney disease; HDL cholesterol |
| Major depressive disorder | rs4497414 | Major depressive disorder; Leisure screen time; Pork consumption |
| Major depressive disorder | rs4936276 | Major depressive disorder; Neuroticism; Sensitivity to environmental stress and adversity |
| Major depressive disorder | rs61914045 | Major depressive disorder; Educational attainment; Highest math class taken (MTAG) |
| Major depressive disorder | rs9529218 | Major depressive disorder; Sum neutrophil eosinophil counts |
| Major depressive disorder | rs9536381 | Major depressive disorder; Multisite chronic pain |
| Major depressive disorder | rs508502 | Major depressive disorder; Height; Central corneal thickness |
| Major depressive disorder | rs1950829 | Major depressive disorder; Depressive symptoms; Well-being spectrum (multivariate analysis) |
| Major depressive disorder | rs754287 | Major depressive disorder; Bipolar disorder (MTAG) |
| Major depressive disorder | rs7152906 | Major depressive disorder; Body fat percentage; General factor of neuroticism |
| Major depressive disorder | rs28541419 | Major depressive disorder; Hip circumference adjusted for BMI |
| Major depressive disorder | rs12919291 | Major depressive disorder; Life satisfaction |
| Major depressive disorder | rs4799949 | Major depressive disorder; Feeling hurt; Worry |
| Major depressive disorder | rs12967143 | Major depressive disorder; Corneal resistance factor (MTAG); Insomnia |
| Major depressive disorder | rs7241572 | Major depressive disorder; Intelligence (MTAG); Educational attainment (years of education) |
| Major depressive disorder | rs1367635 | Major depressive disorder; Putamen volume; Brain region volumes |
| Major depressive disorder | rs13037326 | Major depressive disorder; Systemic lupus erythematosus; Rheumatoid arthritis (ACPA-positive) |
| Schizophrenia | rs11587347 | Schizophrenia; Bipolar disorder (MTAG) |
| Schizophrenia | rs56335113 | Schizophrenia |
| Schizophrenia | rs12129573 | Schizophrenia; Generalized anxiety disorder (mental health questionnaire or predicted); Educational attainment (years of education) |
| Schizophrenia | rs145071536 | Schizophrenia; Body mass index; Self-reported math ability (MTAG) |
| Schizophrenia | rs4653164 | Schizophrenia; Barrett's esophagus x BMI interaction; Height |
| Schizophrenia | rs1892346 | Schizophrenia; Body mass index |
| Schizophrenia | rs1198588 | Schizophrenia; General risk tolerance (MTAG) |
| Schizophrenia | rs12138231 | Schizophrenia; Apolipoprotein A1 levels |
| Schizophrenia | rs7515363 | Schizophrenia; Neutrophil count; C-reactive protein levels |
| Schizophrenia | rs6673880 | Schizophrenia; Body mass index |
| Schizophrenia | rs3795310 | Schizophrenia; Cognitive ability, years of educational attainment or schizophrenia (pleiotropy) |
| Schizophrenia | rs11210892 | Schizophrenia; Intelligence; Household income (MTAG) |
| Schizophrenia | rs11165867 | Schizophrenia; Platelet count; Educational attainment |
| Schizophrenia | rs16851048 | Schizophrenia; N-acetylmethionine sulfoxide levels in elite athletes |
| Schizophrenia | rs1451488 | Schizophrenia; Height |
| Schizophrenia | rs2167378 | Schizophrenia; Height; Humerus length to body height ratio |
| Schizophrenia | rs3791710 | Schizophrenia; Body mass index; Estimated glomerular filtration rate (creatinine) |
| Schizophrenia | rs6715366 | Schizophrenia; Height; Ulcerative colitis |
| Schizophrenia | rs6721531 | Schizophrenia; Metabolite levels; Pulse pressure |
| Schizophrenia | rs13016542 | Schizophrenia; Medication use (diuretics); Educational attainment (years of education) |
| Schizophrenia | rs3739118 | Schizophrenia; Systolic blood pressure |
| Schizophrenia | rs12712510 | Schizophrenia; Externalizing behaviour (multivariate analysis) |
| Schizophrenia | rs7575796 | Schizophrenia; Vertex-wise cortical thickness; |
| Schizophrenia | rs1881046 | Schizophrenia; Educational attainment (years of education) |
| Schizophrenia | rs62183855 | Schizophrenia; Feeling miserable |
| Schizophrenia | rs11693094 | Schizophrenia; Feeling hurt |
| Schizophrenia | rs12151767 | Schizophrenia; Dyslexia |
| Schizophrenia | rs778371 | Schizophrenia; Cortical surface area |
| Schizophrenia | rs13011472 | Schizophrenia; Brain morphology (MOSTest); Intelligence (MTAG) |
| Schizophrenia | rs3770754 | Schizophrenia; Blood protein levels |
| Schizophrenia | rs6546857 | Schizophrenia; Serum metabolite levels |
| Schizophrenia | rs2909457 | Schizophrenia; Intelligence (MTAG) |
| Schizophrenia | rs12489270 | Schizophrenia; Intelligence; Height |
| Schizophrenia | rs6798742 | Schizophrenia; Red cell distribution width |
| Schizophrenia | rs167924 | Schizophrenia; Brain morphology (MOSTest) |
| Schizophrenia | rs1430894 | Schizophrenia; Vertex-wise cortical thickness |
| Schizophrenia | rs6549963 | Schizophrenia; Heel bone mineral density |
| Schizophrenia | rs2710323 | Schizophrenia; BMI (standard GWA); Verbal learning (visual presentation) |
| Schizophrenia | rs17194490 | Schizophrenia; Educational attainment; Self-reported math ability |
| Schizophrenia | rs60135207 | Schizophrenia; Educational attainment |
| Schizophrenia | rs7634476 | Schizophrenia; Triglyceride levels in LDL |
| Schizophrenia | rs9876421 | Schizophrenia; Platelet distribution width |
| Schizophrenia | rs1604060 | Schizophrenia; Educational attainment |
| Schizophrenia | rs308697 | Schizophrenia; Weight; Educational attainment |
| Schizophrenia | rs7647398 | Schizophrenia; Height; Body mass index (MTAG) |
| Schizophrenia | rs35734242 | Schizophrenia; Waist-hip index; Granulocyte count |
| Schizophrenia | rs215412 | Schizophrenia; Ankylosing spondylitis |
| Schizophrenia | rs11941714 | Schizophrenia; Generalized epilepsy; Externalizing behaviour (multivariate analysis) |
| Schizophrenia | rs1427633 | Schizophrenia; Glucose levels (Biocrates platform) |
| Schizophrenia | rs13107325 | Schizophrenia; Hemoglobin levels; Addiction risk factors |
| Schizophrenia | rs2333321 | Schizophrenia; Educational attainment (years of education) |
| Schizophrenia | rs10035564 | Schizophrenia; Refractive error |
| Schizophrenia | rs16867571 | Schizophrenia; Educational attainment (MTAG) |
| Schizophrenia | rs10117 | Schizophrenia; Mean corpuscular hemoglobin |
| Schizophrenia | rs1901512 | Schizophrenia; Waist-to-hip ratio adjusted for BMI |
| Schizophrenia | rs187557 | Schizophrenia; Lobe attachment (rater-scored or self-reported) |
| Schizophrenia | rs72802868 | Schizophrenia; Subjective well-being |
| Schizophrenia | rs11740474 | Schizophrenia; Body mass index |
| Schizophrenia | rs9687282 | Schizophrenia; Whole brain restricted directional diffusion (multivariate analysis) |
| Schizophrenia | rs12652777 | Schizophrenia; Waist-hip ratio |
| Schizophrenia | rs4700418 | Schizophrenia; Intelligence (MTAG) |
| Schizophrenia | rs9454727 | Schizophrenia; Educational attainment (years of education) |
| Schizophrenia | rs2815731 | Schizophrenia; Height |
| Schizophrenia | rs34555420 | Schizophrenia |
| Schizophrenia | rs634940 | Schizophrenia; Univariate microbial feature (family: clostridiales) at the incertae (moist skin) |
| Schizophrenia | rs9461916 | Schizophrenia; Blood pressure (pleiotropy model 1 DBP adjusted for estimated causal effects x SBP) |
| Schizophrenia | rs217336 | Schizophrenia; Height; Body mass index |
| Schizophrenia | rs13195636 | Schizophrenia; Subcortical volume (MOSTest) |
| Schizophrenia | rs1611236 | Schizophrenia; Hip index |
| Schizophrenia | rs7798283 | Schizophrenia; Intelligence |
| Schizophrenia | rs728055 | Schizophrenia; Educational attainment |
| Schizophrenia | rs1914399 | Schizophrenia; Body mass index (MTAG) |
| Schizophrenia | rs13233308 | Schizophrenia; Height; Chickenpox |
| Schizophrenia | rs58120505 | Schizophrenia; Feeling miserable |
| Schizophrenia | rs6943762 | Schizophrenia; Adolescent idiopathic scoliosis |
| Schizophrenia | rs6974218 | Schizophrenia; Attention deficit hyperactivity disorder (time to onset) |
| Schizophrenia | rs35426637 | Schizophrenia; Height |
| Schizophrenia | rs79210963 | Schizophrenia; Systolic blood pressure |
| Schizophrenia | rs2252074 | Schizophrenia; General risk tolerance (MTAG) |
| Schizophrenia | rs1593304 | Schizophrenia; Worry; Body mass index |
| Schizophrenia | rs73229090 | Schizophrenia; Autism spectrum disorder |
| Schizophrenia | rs11136325 | Schizophrenia; Menarche (age at onset) |
| Schizophrenia | rs79445414 | Schizophrenia; Body mass index; Height |
| Schizophrenia | rs6984242 | Schizophrenia; Adult body size; Height |
| Schizophrenia | rs1915019 | Schizophrenia; A body shape index |
| Schizophrenia | rs4129585 | Schizophrenia; F-savour/caloric food liking (derived food-liking factor) |
| Schizophrenia | rs4921741 | Schizophrenia; Calcium levels |
| Schizophrenia | rs10957321 | Schizophrenia; Systolic blood pressure |
| Schizophrenia | rs10103330 | Schizophrenia |
| Schizophrenia | rs10086619 | Schizophrenia; Fibromuscular dysplasia |
| Schizophrenia | rs500102 | Schizophrenia; Height; Cortical surface area |
| Schizophrenia | rs2381411 | Schizophrenia; Acute graft-versus-host disease (stage 2 to 4) (recipient effect) |
| Schizophrenia | rs498591 | Schizophrenia; Self-reported math ability (MTAG) |
| Schizophrenia | rs2078266 | Schizophrenia |
| Schizophrenia | rs505061 | Schizophrenia; Moderate-to-late spontaneous preterm birth |
| Schizophrenia | rs3824451 | Schizophrenia |
| Schizophrenia | rs6482437 | Schizophrenia |
| Schizophrenia | rs61857878 | Schizophrenia; Triacylglycerol (48:2) levels |
| Schizophrenia | rs17731 | Schizophrenia; Hematocrit |
| Schizophrenia | rs12771371 | Schizophrenia; Heel bone mineral density; Height |
| Schizophrenia | rs11191580 | Schizophrenia; Pulse pressure |
| Schizophrenia | rs2514218 | Schizophrenia; Depressed affect |
| Schizophrenia | rs72943392 | Schizophrenia; Cognitive ability, years of educational attainment or schizophrenia (pleiotropy) |
| Schizophrenia | rs7113199 | Schizophrenia; Verbal declarative memory |
| Schizophrenia | rs708228 | Schizophrenia; Blood protein levels |
| Schizophrenia | rs7112616 | Schizophrenia; Autism spectrum disorder or schizophrenia |
| Schizophrenia | rs3802924 | Schizophrenia; Intelligence; Neuroticism |
| Schizophrenia | rs11027839 | Schizophrenia; Plasma neurofilament light levels |
| Schizophrenia | rs4636654 | Schizophrenia; Disruptive behavior (multivariate analysis) |
| Schizophrenia | rs12285419 | Schizophrenia; Headache or migraine |
| Schizophrenia | rs12293670 | Schizophrenia; Blood protein levels; Serum levels of protein ESAM |
| Schizophrenia | rs1860002 | Schizophrenia; Common executive function |
| Schizophrenia | rs10861176 | Schizophrenia; Blood protein levels |
| Schizophrenia | rs4766428 | Schizophrenia; Red blood cell count; Intelligence (MTAG) |
| Schizophrenia | rs12303743 | Schizophrenia; Color vision defects (Tritan) |
| Schizophrenia | rs1615350 | Schizophrenia; Brain morphology (MOSTest) |
| Schizophrenia | rs12833624 | Schizophrenia; Waist circumference adjusted for body mass index |
| Schizophrenia | rs10876446 | Schizophrenia; Height |
| Schizophrenia | rs61937595 | Schizophrenia; Autism spectrum disorder or schizophrenia |
| Schizophrenia | rs6538539 | Schizophrenia; Combined resilience (covariance of educational attainment with residual cognitive resilience) |
| Schizophrenia | rs2455415 | Schizophrenia; Height |
| Schizophrenia | rs12877581 | Schizophrenia; Photic sneeze reflex |
| Schizophrenia | rs9318627 | Schizophrenia; Educational attainment |
| Schizophrenia | rs2332700 | Schizophrenia; Educational attainment (years of education) |
| Schizophrenia | rs10873538 | Schizophrenia; Insomnia |
| Schizophrenia | rs1953205 | Schizophrenia; Cognitive ability, years of educational attainment or schizophrenia (pleiotropy) |
| Schizophrenia | rs12883788 | Schizophrenia; Body mass index in physically active individuals |
| Schizophrenia | rs2999392 | Schizophrenia; Age related hearing loss-related regional glucose metabolism (Cochlear nucleus) |
| Schizophrenia | rs1540840 | Schizophrenia; Common executive function |
| Schizophrenia | rs56205728 | Schizophrenia; G_Roseburia abundance |
| Schizophrenia | rs4779050 | Schizophrenia; General cognitive ability; Intelligence |
| Schizophrenia | rs62018952 | Schizophrenia; Triglyceride levels |
| Schizophrenia | rs35351411 | Schizophrenia; Autism spectrum disorder or schizophrenia |
| Schizophrenia | rs2456020 | Schizophrenia; Airflow obstruction |
| Schizophrenia | rs4702 | Schizophrenia; Diastolic blood pressure |
| Schizophrenia | rs3814883 | Schizophrenia; Waist-hip ratio |
| Schizophrenia | rs149165 | Schizophrenia; Height |
| Schizophrenia | rs4575535 | Schizophrenia; Waist circumference adjusted for body mass index |
| Schizophrenia | rs8055219 | Schizophrenia; Life satisfaction; Positive affect |
| Schizophrenia | rs73292401 | Schizophrenia; Joint mobility (Beighton score) |
| Schizophrenia | rs57433322 | Schizophrenia; Educational attainment (years of education) |
| Schizophrenia | rs2696466 | Schizophrenia; Male puberty timing (early vs. average onset facial hair) |
| Schizophrenia | rs11664298 | Schizophrenia; Intelligence (MTAG) |
| Schizophrenia | rs9304548 | Schizophrenia; Hyperuricemia |
| Schizophrenia | rs4632195 | Schizophrenia; Putamen volume |
| Schizophrenia | rs9636107 | Schizophrenia; Brain shape (segment 2) |
| Schizophrenia | rs72986630 | Schizophrenia; Cognitive ability, years of educational attainment or schizophrenia (pleiotropy) |
| Schizophrenia | rs7251 | Schizophrenia; Average diameter for VLDL particles |
| Schizophrenia | rs1000237 | Schizophrenia; LDL cholesterol |
| Schizophrenia | rs2053079 | Schizophrenia; Adult body size |
| Schizophrenia | rs4812325 | Schizophrenia; Educational attainment (years of education) |
| Schizophrenia | rs11696755 | Schizophrenia; Positive affect |
| Schizophrenia | rs113264400 | Schizophrenia; QT interval; Height |
| Schizophrenia | rs6001259 | Schizophrenia; Neuronal pentraxin receptor levels |
| Schizophrenia | rs132582 | Schizophrenia; Intelligence |
| Schizophrenia | rs5751191 | Schizophrenia; Mean arterial pressure |
| Schizophrenia | rs8138941 | Schizophrenia; Pulse pressure |
| Schizophrenia | rs6010045 | Schizophrenia; Height; Intelligence (MTAG) |

**Supplementary Table S4** | Characteristics of SNPs extracted from exposure data.

| **exposure** | **SNP** | **Position** | **EA** | **OA** | **Beta** | **SE** | **P-Value** | **F-Value** |
| --- | --- | --- | --- | --- | --- | --- | --- | --- |
| Major depressive disorder | rs7551758 | 52274078 | G | T | 0.0283 | 0.0043 | 5.11E-11 | 43.314765 |
| Major depressive disorder | rs2568958 | 72765116 | A | G | 0.0382 | 0.0044 | 2.90E-18 | 75.373967 |
| Major depressive disorder | rs10913112 | 175913828 | T | C | -0.0262 | 0.0045 | 4.53E-09 | 33.898272 |
| Major depressive disorder | rs17641524 | 197704717 | T | C | -0.03 | 0.0053 | 1.50E-08 | 32.039872 |
| Major depressive disorder | rs354155 | 49675276 | C | G | -0.0449 | 0.0075 | 1.75E-09 | 35.840178 |
| Major depressive disorder | rs7538938 | 67132262 | C | T | 0.0251 | 0.0043 | 7.29E-09 | 34.073012 |
| Major depressive disorder | rs4141983 | 18122009 | C | T | -0.0264 | 0.0046 | 9.69E-09 | 32.937618 |
| Major depressive disorder | rs2111592 | 208049581 | A | G | 0.0263 | 0.0046 | 1.35E-08 | 32.688563 |
| Major depressive disorder | rs72948506 | 212618440 | A | G | 0.0265 | 0.0047 | 1.71E-08 | 31.790403 |
| Major depressive disorder | rs35469634 | 158171455 | G | A | -0.0241 | 0.0044 | 3.28E-08 | 30.000517 |
| Major depressive disorder | rs843812 | 61255413 | A | G | 0.0248 | 0.0044 | 1.41E-08 | 31.768595 |
| Major depressive disorder | rs9831648 | 49214303 | T | G | -0.0292 | 0.0052 | 1.59E-08 | 31.532544 |
| Major depressive disorder | rs66511648 | 117515519 | C | T | 0.0297 | 0.0048 | 6.03E-10 | 38.285156 |
| Major depressive disorder | rs76954012 | 115977242 | A | T | 0.0412 | 0.0074 | 2.41E-08 | 30.997809 |
| Major depressive disorder | rs30266 | 103972357 | A | G | 0.0366 | 0.0046 | 1.43E-15 | 63.306238 |
| Major depressive disorder | rs247910 | 87630769 | G | A | 0.0237 | 0.0043 | 4.71E-08 | 30.378042 |
| Major depressive disorder | rs7725715 | 164487555 | A | G | 0.029 | 0.0043 | 1.61E-11 | 45.484045 |
| Major depressive disorder | rs150186873 | 27182377 | C | A | 0.0704 | 0.012 | 4.51E-09 | 34.417778 |
| Major depressive disorder | rs2232423 | 28366151 | G | A | -0.062 | 0.007 | 1.14E-18 | 78.44898 |
| Major depressive disorder | rs9364755 | 165117329 | G | A | 0.0283 | 0.0051 | 3.49E-08 | 30.791619 |
| Major depressive disorder | rs2214123 | 67000001 | G | A | -0.0261 | 0.0045 | 8.56E-09 | 33.64 |
| Major depressive disorder | rs2876520 | 142996618 | G | C | 0.026 | 0.0043 | 2.24E-09 | 36.560303 |
| Major depressive disorder | rs2522831 | 82448100 | C | T | 0.024 | 0.0043 | 2.11E-08 | 31.151974 |
| Major depressive disorder | rs4730387 | 109100414 | A | T | 0.0238 | 0.0043 | 4.12E-08 | 30.634938 |
| Major depressive disorder | rs150346963 | 117625599 | T | C | 0.0283 | 0.0044 | 1.16E-10 | 41.368285 |
| Major depressive disorder | rs3807865 | 12250402 | A | G | 0.031 | 0.0044 | 1.09E-12 | 49.63843 |
| Major depressive disorder | rs10235664 | 2086814 | C | T | -0.027 | 0.0049 | 4.68E-08 | 30.362349 |
| Major depressive disorder | rs59082935 | 38724868 | T | C | 0.0363 | 0.0066 | 3.07E-08 | 30.25 |
| Major depressive disorder | rs62535714 | 37182655 | A | G | 0.0339 | 0.0058 | 4.69E-09 | 34.16201 |
| Major depressive disorder | rs1931388 | 11203149 | G | A | -0.0295 | 0.0044 | 1.68E-11 | 44.95093 |
| Major depressive disorder | rs59283172 | 25232978 | A | G | -0.039 | 0.007 | 2.41E-08 | 31.040816 |
| Major depressive disorder | rs2418449 | 119731359 | C | T | -0.0281 | 0.0048 | 4.25E-09 | 34.271267 |
| Major depressive disorder | rs1021363 | 106610839 | G | A | -0.03 | 0.0045 | 2.29E-11 | 44.444444 |
| Major depressive disorder | rs198457 | 61471678 | T | C | -0.0315 | 0.0056 | 1.90E-08 | 31.640625 |
| Major depressive disorder | rs4497414 | 88756779 | C | T | 0.0291 | 0.0044 | 2.93E-11 | 43.740186 |
| Major depressive disorder | rs4936276 | 113365141 | C | G | 0.0278 | 0.0044 | 3.57E-10 | 39.919421 |
| Major depressive disorder | rs61914045 | 52352301 | A | G | 0.0309 | 0.0054 | 7.96E-09 | 32.743827 |
| Major depressive disorder | rs9529218 | 31790053 | T | C | -0.034 | 0.0054 | 2.23E-10 | 39.643347 |
| Major depressive disorder | rs9536381 | 53860655 | T | C | 0.0255 | 0.0046 | 2.62E-08 | 30.730151 |
| Major depressive disorder | rs508502 | 80921519 | T | C | -0.0264 | 0.0048 | 3.56E-08 | 30.25 |
| Major depressive disorder | rs1950829 | 42097937 | G | A | -0.0297 | 0.0043 | 4.74E-12 | 47.706328 |
| Major depressive disorder | rs754287 | 103997525 | A | T | -0.0289 | 0.0045 | 1.31E-10 | 41.244938 |
| Major depressive disorder | rs7152906 | 75125540 | C | T | 0.0258 | 0.0043 | 1.87E-09 | 36 |
| Major depressive disorder | rs28541419 | 88945878 | G | C | -0.0292 | 0.0052 | 1.76E-08 | 31.532544 |
| Major depressive disorder | rs12919291 | 13800430 | C | G | 0.0327 | 0.0055 | 3.09E-09 | 35.34843 |
| Major depressive disorder | rs4799949 | 35155910 | T | C | -0.0292 | 0.0046 | 1.40E-10 | 40.294896 |
| Major depressive disorder | rs12967143 | 53099012 | C | G | -0.0345 | 0.0047 | 2.53E-13 | 53.881847 |
| Major depressive disorder | rs7241572 | 77580712 | A | G | 0.0323 | 0.0054 | 2.43E-09 | 35.778121 |
| Major depressive disorder | rs1367635 | 50861409 | C | T | 0.0253 | 0.0043 | 4.35E-09 | 34.618172 |
| Major depressive disorder | rs13037326 | 44692598 | T | C | 0.031 | 0.0049 | 2.40E-10 | 40.02499 |
| Schizophrenia | rs11587347 | 239198959 | G | C | 0.103895 | 0.0147 | 1.529E-12 | 49.952201 |
| Schizophrenia | rs56335113 | 30427639 | G | A | -0.0647 | 0.0094 | 6.017E-12 | 47.376861 |
| Schizophrenia | rs12129573 | 73768366 | A | C | 0.077799 | 0.0089 | 2.282E-18 | 76.41392 |
| Schizophrenia | rs145071536 | 243793012 | C | T | 0.085101 | 0.012 | 1.615E-12 | 50.292327 |
| Schizophrenia | rs4653164 | 36627542 | T | C | 0.051104 | 0.0092 | 3.076E-08 | 30.855368 |
| Schizophrenia | rs1892346 | 66331478 | A | T | 0.048403 | 0.0088 | 3.557E-08 | 30.253375 |
| Schizophrenia | rs1198588 | 98552832 | T | A | 0.102598 | 0.0108 | 1.731E-21 | 90.246482 |
| Schizophrenia | rs12138231 | 150115398 | A | T | 0.066995 | 0.0116 | 7.99E-09 | 33.355504 |
| Schizophrenia | rs7515363 | 200414959 | T | C | -0.0535 | 0.0089 | 1.836E-09 | 36.138875 |
| Schizophrenia | rs6673880 | 2373168 | G | A | 0.062301 | 0.0091 | 7.194E-12 | 46.871327 |
| Schizophrenia | rs3795310 | 8431607 | T | C | -0.051 | 0.0087 | 5.75E-09 | 34.360079 |
| Schizophrenia | rs11210892 | 44100084 | A | G | -0.0635 | 0.0091 | 2.678E-12 | 48.693558 |
| Schizophrenia | rs11165867 | 97878068 | T | C | 0.074303 | 0.0116 | 1.3E-10 | 41.029988 |
| Schizophrenia | rs16851048 | 177276006 | C | T | 0.074497 | 0.0107 | 4.149E-12 | 48.474519 |
| Schizophrenia | rs1451488 | 199990107 | G | A | 0.070895 | 0.0087 | 4.472E-16 | 66.403204 |
| Schizophrenia | rs2167378 | 200045258 | T | C | -0.0649 | 0.0087 | 7.305E-14 | 55.644397 |
| Schizophrenia | rs3791710 | 212290048 | C | T | -0.06 | 0.0108 | 3.02E-08 | 30.867593 |
| Schizophrenia | rs6715366 | 2327295 | A | G | 0.054097 | 0.0097 | 2.492E-08 | 31.103274 |
| Schizophrenia | rs6721531 | 79426855 | T | A | -0.0517 | 0.0091 | 1.468E-08 | 32.277632 |
| Schizophrenia | rs13016542 | 145183851 | C | T | -0.0883 | 0.0129 | 8.281E-12 | 46.857633 |
| Schizophrenia | rs3739118 | 201253769 | A | G | -0.057 | 0.0095 | 2.364E-09 | 36.005053 |
| Schizophrenia | rs12712510 | 22749726 | C | T | -0.0574 | 0.0087 | 5.144E-11 | 43.53057 |
| Schizophrenia | rs7575796 | 97746526 | G | A | -0.0963 | 0.0172 | 2.065E-08 | 31.347369 |
| Schizophrenia | rs1881046 | 156835793 | T | G | -0.0507 | 0.0092 | 3.392E-08 | 30.372798 |
| Schizophrenia | rs62183855 | 172956449 | C | A | -0.0661 | 0.0111 | 2.658E-09 | 35.457948 |
| Schizophrenia | rs11693094 | 185601420 | T | C | -0.0544 | 0.0087 | 4.292E-10 | 39.10274 |
| Schizophrenia | rs12151767 | 198274929 | A | G | -0.0611 | 0.0086 | 1.307E-12 | 50.483504 |
| Schizophrenia | rs778371 | 233743109 | G | A | 0.080603 | 0.0095 | 1.495E-17 | 71.987008 |
| Schizophrenia | rs13011472 | 57961602 | G | C | 0.070401 | 0.0087 | 4.282E-16 | 65.48158 |
| Schizophrenia | rs3770754 | 37575381 | G | C | -0.0529 | 0.0091 | 5.352E-09 | 33.788031 |
| Schizophrenia | rs6546857 | 73837955 | G | A | 0.060398 | 0.0102 | 2.744E-09 | 35.062421 |
| Schizophrenia | rs2909457 | 162845855 | A | G | -0.049 | 0.0087 | 1.482E-08 | 31.721107 |
| Schizophrenia | rs12489270 | 16875823 | C | T | 0.057905 | 0.0089 | 7.468E-11 | 42.32979 |
| Schizophrenia | rs6798742 | 63903759 | G | A | 0.061099 | 0.0093 | 4.569E-11 | 43.162216 |
| Schizophrenia | rs167924 | 107379837 | G | A | 0.050199 | 0.009 | 2.339E-08 | 31.110613 |
| Schizophrenia | rs1430894 | 17868759 | T | C | 0.053295 | 0.0086 | 6.154E-10 | 38.404394 |
| Schizophrenia | rs6549963 | 30044778 | C | T | -0.0483 | 0.0088 | 4.308E-08 | 30.130493 |
| Schizophrenia | rs2710323 | 52815905 | C | T | -0.0784 | 0.0086 | 1.229E-19 | 83.115873 |
| Schizophrenia | rs17194490 | 2547786 | T | G | 0.078199 | 0.0116 | 1.795E-11 | 45.445498 |
| Schizophrenia | rs60135207 | 71563777 | T | G | -0.0496 | 0.0088 | 1.526E-08 | 31.767826 |
| Schizophrenia | rs7634476 | 136398387 | G | A | 0.057703 | 0.0088 | 5.463E-11 | 42.996782 |
| Schizophrenia | rs9876421 | 36848316 | T | C | 0.062503 | 0.0092 | 9.192E-12 | 46.156221 |
| Schizophrenia | rs1604060 | 117772036 | G | A | 0.077205 | 0.014 | 3.241E-08 | 30.411365 |
| Schizophrenia | rs308697 | 161487491 | A | C | -0.0501 | 0.0087 | 8.832E-09 | 33.166478 |
| Schizophrenia | rs7647398 | 180733150 | T | C | -0.0775 | 0.0109 | 1.074E-12 | 50.550665 |
| Schizophrenia | rs35734242 | 706700 | C | T | 0.050704 | 0.0089 | 1.368E-08 | 32.456705 |
| Schizophrenia | rs215412 | 23423586 | A | G | 0.057703 | 0.0091 | 2.692E-10 | 40.20856 |
| Schizophrenia | rs11941714 | 31202669 | A | G | -0.0516 | 0.0093 | 3.073E-08 | 30.779469 |
| Schizophrenia | rs1427633 | 167974142 | C | G | -0.0483 | 0.0088 | 4.097E-08 | 30.130493 |
| Schizophrenia | rs13107325 | 103188709 | T | C | 0.158703 | 0.0168 | 2.9E-21 | 89.238387 |
| Schizophrenia | rs2333321 | 176859992 | G | A | -0.0712 | 0.0105 | 1.248E-11 | 45.986223 |
| Schizophrenia | rs10035564 | 45252500 | G | A | 0.066802 | 0.0092 | 4.38E-13 | 52.724015 |
| Schizophrenia | rs16867571 | 88743219 | G | A | -0.0657 | 0.0104 | 2.676E-10 | 39.912629 |
| Schizophrenia | rs10117 | 137892170 | A | G | -0.055 | 0.0088 | 4.659E-10 | 39.061648 |
| Schizophrenia | rs1901512 | 101723875 | C | T | -0.0584 | 0.0094 | 5.723E-10 | 38.599783 |
| Schizophrenia | rs187557 | 106766340 | T | C | -0.0667 | 0.0119 | 2.029E-08 | 31.412351 |
| Schizophrenia | rs72802868 | 152235215 | T | G | -0.0692 | 0.0096 | 4.554E-13 | 51.959319 |
| Schizophrenia | rs11740474 | 153680747 | T | A | 0.053696 | 0.0088 | 1.128E-09 | 37.232462 |
| Schizophrenia | rs9687282 | 139065988 | G | T | 0.052599 | 0.0091 | 7.328E-09 | 33.410178 |
| Schizophrenia | rs12652777 | 155775075 | C | T | -0.0488 | 0.0086 | 1.523E-08 | 32.198631 |
| Schizophrenia | rs4700418 | 60621839 | G | C | 0.070197 | 0.0087 | 5.367E-16 | 65.103011 |
| Schizophrenia | rs9454727 | 70003389 | G | A | -0.0544 | 0.0098 | 3.35E-08 | 30.817226 |
| Schizophrenia | rs2815731 | 73155285 | A | C | -0.06 | 0.0091 | 4.388E-11 | 43.477793 |
| Schizophrenia | rs34555420 | 26090270 | T | G | -0.1687 | 0.0173 | 1.537E-22 | 95.086172 |
| Schizophrenia | rs634940 | 93077500 | T | G | 0.066396 | 0.0099 | 1.784E-11 | 44.979649 |
| Schizophrenia | rs9461916 | 33796794 | C | T | 0.053295 | 0.0088 | 1.641E-09 | 36.678577 |
| Schizophrenia | rs217336 | 84341803 | A | C | -0.0503 | 0.0087 | 8.051E-09 | 33.431391 |
| Schizophrenia | rs13195636 | 27509493 | C | A | -0.2105 | 0.0159 | 6.546E-40 | 175.27762 |
| Schizophrenia | rs1611236 | 29748690 | A | G | -0.0551 | 0.0096 | 8.474E-09 | 32.947002 |
| Schizophrenia | rs7798283 | 133128127 | G | T | -0.074 | 0.0134 | 3.487E-08 | 30.499243 |
| Schizophrenia | rs728055 | 137072531 | A | T | -0.0674 | 0.009 | 8.849E-14 | 56.078298 |
| Schizophrenia | rs1914399 | 71770973 | G | C | -0.0491 | 0.0087 | 1.401E-08 | 31.856812 |
| Schizophrenia | rs13233308 | 87244960 | T | C | -0.0487 | 0.0086 | 1.749E-08 | 32.072993 |
| Schizophrenia | rs58120505 | 2029867 | C | T | -0.0896 | 0.0088 | 2.235E-24 | 103.67636 |
| Schizophrenia | rs6943762 | 86403263 | C | T | -0.1051 | 0.0132 | 1.569E-15 | 63.392961 |
| Schizophrenia | rs6974218 | 110056000 | C | A | -0.0549 | 0.0089 | 6.804E-10 | 38.044363 |
| Schizophrenia | rs35426637 | 110988593 | T | G | -0.0623 | 0.0093 | 2.151E-11 | 44.873432 |
| Schizophrenia | rs79210963 | 24717969 | C | T | 0.085602 | 0.0137 | 4.142E-10 | 39.041061 |
| Schizophrenia | rs2252074 | 104594253 | G | T | 0.068504 | 0.0088 | 6.192E-15 | 60.598617 |
| Schizophrenia | rs1593304 | 131619847 | G | A | 0.064101 | 0.0111 | 7.448E-09 | 33.349376 |
| Schizophrenia | rs73229090 | 27442127 | A | C | -0.1026 | 0.0142 | 4.337E-13 | 52.207748 |
| Schizophrenia | rs11136325 | 144870701 | A | G | -0.0538 | 0.0091 | 3.046E-09 | 34.948496 |
| Schizophrenia | rs79445414 | 33863561 | C | T | 0.1234 | 0.0222 | 2.798E-08 | 30.897573 |
| Schizophrenia | rs6984242 | 60700469 | A | G | -0.0547 | 0.0087 | 3.855E-10 | 39.525791 |
| Schizophrenia | rs1915019 | 89283689 | G | A | -0.0571 | 0.0098 | 6.574E-09 | 33.946556 |
| Schizophrenia | rs4129585 | 143312933 | C | A | -0.075 | 0.0087 | 5.109E-18 | 74.30876 |
| Schizophrenia | rs4921741 | 17070926 | G | A | 0.055999 | 0.0098 | 1.205E-08 | 32.652012 |
| Schizophrenia | rs10957321 | 65605878 | A | G | 0.047595 | 0.0086 | 3.485E-08 | 30.628374 |
| Schizophrenia | rs10103330 | 4180090 | A | T | 0.067305 | 0.0108 | 5.067E-10 | 38.837132 |
| Schizophrenia | rs10086619 | 111580570 | G | A | 0.072205 | 0.0116 | 4.968E-10 | 38.745473 |
| Schizophrenia | rs500102 | 77358745 | C | T | -0.0517 | 0.0088 | 4.871E-09 | 34.515892 |
| Schizophrenia | rs2381411 | 36319928 | C | T | 0.050399 | 0.0088 | 1.25E-08 | 32.800351 |
| Schizophrenia | rs498591 | 14509105 | T | A | 0.072495 | 0.0121 | 2.114E-09 | 35.896339 |
| Schizophrenia | rs2078266 | 138378856 | G | A | -0.0696 | 0.0126 | 2.941E-08 | 30.513085 |
| Schizophrenia | rs505061 | 22767164 | A | C | 0.053496 | 0.0086 | 5.804E-10 | 38.693752 |
| Schizophrenia | rs3824451 | 101071522 | C | T | 0.065595 | 0.0118 | 2.539E-08 | 30.901445 |
| Schizophrenia | rs6482437 | 18726326 | C | A | 0.098904 | 0.0142 | 3.326E-12 | 48.511814 |
| Schizophrenia | rs61857878 | 92789488 | T | A | -0.0601 | 0.0102 | 4.441E-09 | 34.714509 |
| Schizophrenia | rs17731 | 3821561 | A | G | 0.052399 | 0.0089 | 4.37E-09 | 34.663252 |
| Schizophrenia | rs12771371 | 54063083 | A | G | -0.0524 | 0.0093 | 1.94E-08 | 31.749832 |
| Schizophrenia | rs11191580 | 104906211 | C | T | -0.1317 | 0.0155 | 1.772E-17 | 72.198461 |
| Schizophrenia | rs2514218 | 113392994 | T | C | -0.0705 | 0.0092 | 1.348E-14 | 58.715072 |
| Schizophrenia | rs72943392 | 81178838 | C | G | 0.053496 | 0.0096 | 2.393E-08 | 31.052408 |
| Schizophrenia | rs7113199 | 134247187 | C | A | -0.0523 | 0.0094 | 2.801E-08 | 30.954189 |
| Schizophrenia | rs708228 | 57585662 | T | C | 0.0528 | 0.0091 | 6.556E-09 | 33.665117 |
| Schizophrenia | rs7112616 | 130805334 | C | T | -0.0522 | 0.0086 | 1.517E-09 | 36.846876 |
| Schizophrenia | rs3802924 | 133827733 | C | A | -0.0736 | 0.0108 | 9.583E-12 | 46.446244 |
| Schizophrenia | rs11027839 | 24389235 | C | A | 0.051504 | 0.0086 | 2.398E-09 | 35.865893 |
| Schizophrenia | rs4636654 | 28642653 | A | G | -0.0483 | 0.0089 | 4.892E-08 | 29.457207 |
| Schizophrenia | rs12285419 | 46343189 | A | C | 0.084905 | 0.011 | 1.048E-14 | 59.576646 |
| Schizophrenia | rs12293670 | 124612932 | G | A | -0.0705 | 0.0092 | 1.557E-14 | 58.715072 |
| Schizophrenia | rs1860002 | 2413803 | T | C | -0.0838 | 0.0087 | 1.039E-21 | 92.776088 |
| Schizophrenia | rs10861176 | 104631552 | A | G | 0.055502 | 0.0098 | 1.588E-08 | 32.075001 |
| Schizophrenia | rs4766428 | 110723245 | T | C | 0.075004 | 0.0089 | 3.931E-17 | 71.020957 |
| Schizophrenia | rs12303743 | 72259954 | C | G | 0.087499 | 0.0145 | 1.59E-09 | 36.413983 |
| Schizophrenia | rs1615350 | 123650335 | T | C | -0.0736 | 0.0098 | 4.918E-14 | 56.408683 |
| Schizophrenia | rs12833624 | 124476873 | T | C | 0.050199 | 0.009 | 2.773E-08 | 31.110613 |
| Schizophrenia | rs10876446 | 53760710 | C | G | 0.054002 | 0.0094 | 1.026E-08 | 33.004047 |
| Schizophrenia | rs61937595 | 57682956 | T | C | -0.1301 | 0.0162 | 1.147E-15 | 64.492797 |
| Schizophrenia | rs6538539 | 95195293 | T | G | -0.0568 | 0.0086 | 4.428E-11 | 43.615427 |
| Schizophrenia | rs2455415 | 38860697 | T | C | 0.049495 | 0.0088 | 1.69E-08 | 31.634105 |
| Schizophrenia | rs12877581 | 74325499 | C | G | 0.059601 | 0.0099 | 1.803E-09 | 36.244535 |
| Schizophrenia | rs9318627 | 79930079 | C | A | -0.0612 | 0.0088 | 4.352E-12 | 48.36349 |
| Schizophrenia | rs2332700 | 72417326 | G | C | -0.0751 | 0.0099 | 3.875E-14 | 57.542492 |
| Schizophrenia | rs10873538 | 104255569 | G | T | 0.066503 | 0.0091 | 3.01E-13 | 53.407346 |
| Schizophrenia | rs1953205 | 30300361 | A | T | 0.049905 | 0.0089 | 2.215E-08 | 31.441599 |
| Schizophrenia | rs12883788 | 33303540 | T | C | 0.061301 | 0.0087 | 1.862E-12 | 49.647574 |
| Schizophrenia | rs2999392 | 51655145 | T | C | 0.051799 | 0.0094 | 3.05E-08 | 30.36561 |
| Schizophrenia | rs1540840 | 99733384 | C | G | -0.0557 | 0.0093 | 2.212E-09 | 35.870568 |
| Schizophrenia | rs56205728 | 40567237 | A | G | 0.063004 | 0.0097 | 1.007E-10 | 42.187971 |
| Schizophrenia | rs4779050 | 83368738 | G | T | -0.058 | 0.0089 | 7.274E-11 | 42.462502 |
| Schizophrenia | rs62018952 | 44080737 | C | T | 0.058403 | 0.0097 | 1.941E-09 | 36.2512 |
| Schizophrenia | rs35351411 | 61872197 | C | A | 0.063504 | 0.0087 | 2.211E-13 | 53.280603 |
| Schizophrenia | rs2456020 | 78868398 | T | C | -0.0816 | 0.0102 | 1.125E-15 | 63.99749 |
| Schizophrenia | rs4702 | 91426560 | A | G | -0.0843 | 0.0089 | 2.794E-21 | 89.726447 |
| Schizophrenia | rs3814883 | 29994922 | T | C | -0.0671 | 0.0087 | 1.576E-14 | 59.480795 |
| Schizophrenia | rs149165 | 58659307 | G | T | -0.0482 | 0.0087 | 3.005E-08 | 30.69351 |
| Schizophrenia | rs4575535 | 89559297 | G | A | 0.055798 | 0.0096 | 5.77E-09 | 33.782977 |
| Schizophrenia | rs8055219 | 13753384 | A | G | 0.066503 | 0.0101 | 5.692E-11 | 43.355184 |
| Schizophrenia | rs73292401 | 12875908 | A | T | 0.067605 | 0.0109 | 5.48E-10 | 38.467877 |
| Schizophrenia | rs57433322 | 19141582 | G | C | -0.0831 | 0.0139 | 1.992E-09 | 35.741129 |
| Schizophrenia | rs2696466 | 44289832 | G | A | -0.0612 | 0.0092 | 2.637E-11 | 44.249393 |
| Schizophrenia | rs11664298 | 77578986 | A | G | 0.0774 | 0.0108 | 8.935E-13 | 51.360448 |
| Schizophrenia | rs9304548 | 27500959 | A | C | -0.0567 | 0.01 | 1.594E-08 | 32.150714 |
| Schizophrenia | rs4632195 | 50746748 | T | C | 0.047196 | 0.0086 | 4.586E-08 | 30.117633 |
| Schizophrenia | rs9636107 | 53200117 | G | A | 0.069897 | 0.0086 | 5.114E-16 | 66.057012 |
| Schizophrenia | rs72986630 | 11849736 | T | C | 0.112296 | 0.0179 | 3.594E-10 | 39.357048 |
| Schizophrenia | rs7251 | 50162909 | G | C | -0.0641 | 0.0094 | 8.293E-12 | 46.502098 |
| Schizophrenia | rs1000237 | 19518316 | A | T | 0.073205 | 0.0089 | 2.8E-16 | 67.6558 |
| Schizophrenia | rs2053079 | 30987423 | G | A | 0.059899 | 0.0101 | 3.007E-09 | 35.171476 |
| Schizophrenia | rs4812325 | 37485458 | A | G | 0.071904 | 0.0089 | 8.96E-16 | 65.272238 |
| Schizophrenia | rs11696755 | 48105317 | C | T | 0.063696 | 0.011 | 7.26E-09 | 33.530627 |
| Schizophrenia | rs113264400 | 62150128 | C | T | 0.112296 | 0.0202 | 2.865E-08 | 30.904793 |
| Schizophrenia | rs6001259 | 39247750 | T | C | 0.1915 | 0.0348 | 3.696E-08 | 30.281617 |
| Schizophrenia | rs132582 | 39988175 | T | C | -0.051 | 0.0086 | 3.259E-09 | 35.163797 |
| Schizophrenia | rs5751191 | 42370991 | C | T | 0.065595 | 0.0086 | 3.004E-14 | 58.176273 |
| Schizophrenia | rs8138941 | 50290678 | A | G | 0.058095 | 0.0106 | 4.464E-08 | 30.037948 |
| Schizophrenia | rs6010045 | 51103091 | C | T | 0.0549 | 0.0095 | 7.445E-09 | 33.395989 |
| Smoking initiation | rs3001723 | 44037685 | A | G | 0.033512 | 0.0039 | 8.121E-18 | 73.900095 |
| Smoking initiation | rs7555507 | 73766037 | T | C | -0.02414 | 0.00356 | 1.14E-11 | 46.099855 |
| Smoking initiation | rs6669839 | 50625979 | T | C | 0.026004 | 0.0044 | 3.36E-09 | 34.999923 |
| Smoking initiation | rs12042107 | 91196176 | C | T | -0.02228 | 0.00357 | 4.22E-10 | 38.999993 |
| Smoking initiation | rs2186122 | 66470206 | T | A | 0.026057 | 0.00359 | 3.61E-13 | 52.799938 |
| Smoking initiation | rs301805 | 8481016 | G | T | 0.021468 | 0.00361 | 2.8E-09 | 35.299901 |
| Smoking initiation | rs12025237 | 154205120 | C | A | -0.033 | 0.00534 | 6.52E-10 | 38.199931 |
| Smoking initiation | rs2050586 | 87905828 | C | G | -0.02055 | 0.00371 | 3E-08 | 30.700053 |
| Smoking initiation | rs2046850 | 210304319 | T | C | -0.02481 | 0.00448 | 3.03E-08 | 30.700144 |
| Smoking initiation | rs6728726 | 623976 | C | T | 0.035449 | 0.00473 | 6.73E-14 | 56.100109 |
| Smoking initiation | rs78411160 | 58171220 | C | A | 0.020536 | 0.00366 | 2.03E-08 | 31.499847 |
| Smoking initiation | rs6433897 | 182034448 | C | T | 0.022448 | 0.00406 | 3.16E-08 | 30.600151 |
| Smoking initiation | rs266047 | 104088751 | A | G | -0.03051 | 0.00374 | 3.36E-16 | 66.599709 |
| Smoking initiation | rs4674993 | 226332033 | G | A | -0.02521 | 0.00444 | 1.32E-08 | 32.299902 |
| Smoking initiation | rs578584 | 45143175 | T | A | 0.02868 | 0.0036 | 1.5E-15 | 63.599945 |
| Smoking initiation | rs35702515 | 137542847 | T | G | 0.025244 | 0.00423 | 2.43E-09 | 35.600127 |
| Smoking initiation | rs13030994 | 146143090 | A | G | 0.036093 | 0.00356 | 3.56E-24 | 102.99998 |
| Smoking initiation | rs12474587 | 162802993 | T | G | 0.027633 | 0.00358 | 1.25E-14 | 59.500323 |
| Smoking initiation | rs2107300 | 200937901 | G | C | -0.0272 | 0.00493 | 3.27E-08 | 30.499946 |
| Smoking initiation | rs7585579 | 60024857 | G | C | 0.0224 | 0.00373 | 1.88E-09 | 36.099852 |
| Smoking initiation | rs1445649 | 155682556 | C | T | 0.023993 | 0.00356 | 1.68E-11 | 45.299798 |
| Smoking initiation | rs6788098 | 85624131 | T | A | -0.03135 | 0.00369 | 1.91E-17 | 72.200124 |
| Smoking initiation | rs12632110 | 50224225 | G | A | -0.02338 | 0.00375 | 4.78E-10 | 38.799981 |
| Smoking initiation | rs11712680 | 75009019 | C | A | -0.02705 | 0.00458 | 3.51E-09 | 34.89991 |
| Smoking initiation | rs1154693 | 117804154 | G | A | 0.032622 | 0.00491 | 3.12E-11 | 44.100131 |
| Smoking initiation | rs66680800 | 85985324 | T | G | -0.02027 | 0.00365 | 2.83E-08 | 30.800148 |
| Smoking initiation | rs1869243 | 5724536 | C | T | 0.019741 | 0.00356 | 2.97E-08 | 30.699964 |
| Smoking initiation | rs9835772 | 85766025 | T | A | 0.024047 | 0.00414 | 6.32E-09 | 33.700105 |
| Smoking initiation | rs962625 | 28473524 | G | A | 0.023718 | 0.00404 | 4.37E-09 | 34.500128 |
| Smoking initiation | rs993700 | 67825894 | C | T | -0.02593 | 0.00429 | 1.53E-09 | 36.500053 |
| Smoking initiation | rs13145728 | 140927812 | C | G | -0.02325 | 0.00366 | 2.14E-10 | 40.299963 |
| Smoking initiation | rs10001365 | 147797214 | A | G | -0.02499 | 0.00364 | 6.65E-12 | 47.100175 |
| Smoking initiation | rs1160685 | 94052854 | G | C | 0.020772 | 0.00359 | 7.2E-09 | 33.49989 |
| Smoking initiation | rs6893752 | 60374912 | G | A | -0.0241 | 0.00407 | 3.25E-09 | 34.999983 |
| Smoking initiation | rs12186738 | 103816655 | T | G | -0.03326 | 0.00502 | 3.42E-11 | 43.899919 |
| Smoking initiation | rs1385108 | 154839646 | T | C | 0.024662 | 0.00416 | 3E-09 | 35.199978 |
| Smoking initiation | rs4044321 | 166989513 | G | A | -0.02784 | 0.00371 | 6.08E-14 | 56.299936 |
| Smoking initiation | rs4352629 | 87756821 | T | C | -0.02753 | 0.00357 | 1.22E-14 | 59.499742 |
| Smoking initiation | rs72789632 | 106834363 | T | C | -0.03289 | 0.00529 | 5.02E-10 | 38.700022 |
| Smoking initiation | rs9401770 | 98748008 | A | G | 0.027731 | 0.00399 | 3.47E-12 | 48.400191 |
| Smoking initiation | rs222449 | 52916062 | T | A | -0.02532 | 0.00443 | 1.08E-08 | 32.699979 |
| Smoking initiation | rs3800227 | 108994161 | G | A | 0.022812 | 0.00406 | 1.93E-08 | 31.600007 |
| Smoking initiation | rs10498846 | 67405337 | T | C | 0.02061 | 0.00356 | 6.62E-09 | 33.600086 |
| Smoking initiation | rs240963 | 111644332 | C | T | -0.04104 | 0.00484 | 2.16E-17 | 72.000266 |
| Smoking initiation | rs12333760 | 99185406 | C | T | -0.02905 | 0.0048 | 1.44E-09 | 36.600021 |
| Smoking initiation | rs10233018 | 117523709 | G | A | 0.027069 | 0.00356 | 2.75E-14 | 57.899736 |
| Smoking initiation | rs10279261 | 133589846 | A | G | -0.02142 | 0.00366 | 5E-09 | 34.200189 |
| Smoking initiation | rs10260968 | 1889773 | A | G | -0.02032 | 0.00361 | 1.75E-08 | 31.69999 |
| Smoking initiation | rs12112638 | 69735251 | G | A | -0.02453 | 0.00404 | 1.34E-09 | 36.800025 |
| Smoking initiation | rs4236259 | 1708080 | G | T | -0.02477 | 0.00356 | 3.35E-12 | 48.499912 |
| Smoking initiation | rs2140114 | 3407568 | T | C | -0.02326 | 0.00373 | 4.7E-10 | 38.800072 |
| Smoking initiation | rs3801289 | 96638267 | C | A | -0.02206 | 0.00374 | 3.74E-09 | 34.8001 |
| Smoking initiation | rs1565735 | 27426077 | A | T | -0.03762 | 0.00446 | 3.42E-17 | 71.099828 |
| Smoking initiation | rs1899896 | 93201036 | T | C | 0.026448 | 0.00389 | 1.04E-11 | 46.299893 |
| Smoking initiation | rs13261666 | 59814666 | T | G | -0.02689 | 0.00356 | 3.9E-14 | 57.200115 |
| Smoking initiation | rs12545053 | 65073605 | G | A | 0.020281 | 0.00364 | 2.43E-08 | 31.099971 |
| Smoking initiation | rs2631024 | 91995577 | G | A | -0.02296 | 0.00403 | 1.18E-08 | 32.500158 |
| Smoking initiation | rs4543592 | 3014254 | C | T | 0.021931 | 0.00356 | 7.46E-10 | 37.899859 |
| Smoking initiation | rs2378662 | 86707289 | A | G | 0.020948 | 0.00357 | 4.16E-09 | 34.500163 |
| Smoking initiation | rs10114490 | 11070165 | A | G | -0.02551 | 0.00453 | 1.81E-08 | 31.700062 |
| Smoking initiation | rs10905461 | 8803551 | C | T | -0.02396 | 0.00415 | 7.35E-09 | 33.400065 |
| Smoking initiation | rs7921378 | 63674885 | C | G | -0.02546 | 0.00356 | 8.26E-13 | 51.200206 |
| Smoking initiation | rs12356821 | 104563808 | C | G | 0.03937 | 0.00505 | 6.27E-15 | 60.799903 |
| Smoking initiation | rs10159545 | 21766969 | G | C | 0.02625 | 0.00373 | 1.84E-12 | 49.599833 |
| Smoking initiation | rs9423279 | 125680419 | G | C | -0.02051 | 0.00371 | 3.21E-08 | 30.600026 |
| Smoking initiation | rs7938812 | 112911004 | G | T | 0.043791 | 0.00364 | 2.71E-33 | 144.99983 |
| Smoking initiation | rs6265 | 27679916 | T | C | -0.03179 | 0.00458 | 3.77E-12 | 48.199973 |
| Smoking initiation | rs7929518 | 85980958 | G | A | 0.024238 | 0.00428 | 1.56E-08 | 32.000008 |
| Smoking initiation | rs4523689 | 7950797 | G | A | -0.02061 | 0.00364 | 1.55E-08 | 31.999975 |
| Smoking initiation | rs11057005 | 16748721 | G | A | -0.02093 | 0.00358 | 4.85E-09 | 34.200005 |
| Smoking initiation | rs4759228 | 56508409 | C | G | -0.02169 | 0.00393 | 3.58E-08 | 30.400012 |
| Smoking initiation | rs7969559 | 69655167 | G | A | -0.02438 | 0.00396 | 7.31E-10 | 37.899956 |
| Smoking initiation | rs1971318 | 121389500 | T | C | 0.028507 | 0.00493 | 7.06E-09 | 33.49998 |
| Smoking initiation | rs7322872 | 100548329 | T | C | -0.02557 | 0.00433 | 3.58E-09 | 34.800021 |
| Smoking initiation | rs3904512 | 38357471 | A | G | -0.02116 | 0.00358 | 3.23E-09 | 35.000135 |
| Smoking initiation | rs9540729 | 66947124 | T | A | -0.01955 | 0.00356 | 3.82E-08 | 30.19995 |
| Smoking initiation | rs76214862 | 29500130 | C | A | -0.02499 | 0.00455 | 3.99E-08 | 30.200009 |
| Smoking initiation | rs12441907 | 83922387 | A | C | -0.02921 | 0.00452 | 1.06E-10 | 41.70011 |
| Smoking initiation | rs1435741 | 47935843 | A | G | 0.029415 | 0.00359 | 2.64E-16 | 67.099911 |
| Smoking initiation | rs4785836 | 65604652 | C | T | -0.02047 | 0.00366 | 2.26E-08 | 31.299837 |
| Smoking initiation | rs7197072 | 717085 | T | C | -0.02477 | 0.00417 | 2.77E-09 | 35.299892 |
| Smoking initiation | rs1050847 | 87443734 | T | C | -0.02162 | 0.00359 | 1.67E-09 | 36.299943 |
| Smoking initiation | rs4781977 | 17572674 | C | T | -0.02387 | 0.00436 | 4.54E-08 | 29.900167 |
| Smoking initiation | rs11078713 | 7795972 | G | A | -0.02017 | 0.00361 | 2.23E-08 | 31.300028 |
| Smoking initiation | rs7224742 | 30657058 | T | C | -0.02071 | 0.00366 | 1.43E-08 | 32.100012 |
| Smoking initiation | rs11658881 | 2072949 | G | A | 0.020136 | 0.00361 | 2.43E-08 | 31.099992 |
| Smoking initiation | rs6508144 | 50026142 | G | C | -0.02069 | 0.00359 | 7.97E-09 | 33.299866 |
| Smoking initiation | rs11872397 | 72535282 | A | G | -0.02477 | 0.00409 | 1.43E-09 | 36.599963 |
| Smoking initiation | rs72896886 | 42632652 | C | G | -0.02689 | 0.00484 | 2.75E-08 | 30.900067 |
| Smoking initiation | rs76608582 | 4474725 | A | C | -0.04956 | 0.00826 | 1.94E-09 | 36.000029 |
| Smoking initiation | rs1555445 | 31175258 | T | A | 0.022555 | 0.00382 | 3.65E-09 | 34.79994 |
| Smoking initiation | rs117143374 | 40555561 | C | T | 0.02929 | 0.00527 | 2.76E-08 | 30.90001 |
| Smoking initiation | rs134529 | 28781758 | C | T | -0.01998 | 0.00366 | 4.85E-08 | 29.80009 |
| Pack years of smoking | rs7581162 | 60704484 | A | T | 0.019841 | 0.00353 | 1.9E-08 | 31.564832 |
| Pack years of smoking | rs9358909 | 26153545 | C | G | -0.02994 | 0.00449 | 2.5E-11 | 44.521835 |
| Pack years of smoking | rs10226228 | 32315613 | G | A | 0.02661 | 0.0036 | 1.5E-13 | 54.533377 |
| Pack years of smoking | rs6987704 | 42547623 | C | T | 0.026113 | 0.00431 | 1.4E-09 | 36.625165 |
| Pack years of smoking | rs3025360 | 136481205 | A | G | 0.046827 | 0.00539 | 3.7E-18 | 75.469094 |
| Pack years of smoking | rs7933830 | 16377119 | T | C | 0.021152 | 0.00377 | 1.9E-08 | 31.558101 |
| Pack years of smoking | rs2026174 | 112179953 | T | C | 0.022774 | 0.00348 | 5.901E-11 | 42.854217 |
| Pack years of smoking | rs4900590 | 104146421 | T | C | 0.022111 | 0.00371 | 2.5E-09 | 35.544201 |
| Pack years of smoking | rs8042849 | 78817929 | T | C | -0.06843 | 0.00365 | 2.1E-78 | 351.41663 |
| Pack years of smoking | rs2316205 | 41346768 | C | T | 0.02611 | 0.00349 | 7.199E-14 | 55.999535 |
| Pack years of smoking | rs45497800 | 61991833 | T | C | 0.042802 | 0.00624 | 6.8E-12 | 47.083435 |
| Cigarettes smoked per day | rs149905136 | 201207340 | C | T | -0.9219 | 0.1868 | 7.957E-07 | 24.356444 |
| Cigarettes smoked per day | rs7513154 | 70056683 | G | A | -1.2386 | 0.2788 | 8.911E-06 | 19.736794 |
| Cigarettes smoked per day | rs547860 | 33398985 | G | T | -0.3564 | 0.0785 | 5.633E-06 | 20.612757 |
| Cigarettes smoked per day | rs11209132 | 68106279 | G | A | -0.3334 | 0.0708 | 2.512E-06 | 22.175053 |
| Cigarettes smoked per day | rs148139429 | 15767469 | G | A | -1.072 | 0.2343 | 4.762E-06 | 20.933652 |
| Cigarettes smoked per day | rs517533 | 120229170 | G | T | 0.2938 | 0.0653 | 6.839E-06 | 20.243109 |
| Cigarettes smoked per day | rs570081102 | 217627885 | T | C | -0.7791 | 0.1666 | 2.906E-06 | 21.869377 |
| Cigarettes smoked per day | rs77621128 | 80142909 | G | A | 0.5353 | 0.1114 | 1.555E-06 | 23.090009 |
| Cigarettes smoked per day | rs116515084 | 4484271 | A | G | 1.1881 | 0.2642 | 6.894E-06 | 20.222757 |
| Cigarettes smoked per day | rs55717031 | 138848505 | T | G | -0.3088 | 0.0695 | 8.77E-06 | 19.741719 |
| Cigarettes smoked per day | rs112957737 | 41313297 | G | A | -0.9559 | 0.2074 | 4.037E-06 | 21.242588 |
| Cigarettes smoked per day | rs116544150 | 120988405 | A | C | -1.1575 | 0.2066 | 2.106E-08 | 31.389281 |
| Cigarettes smoked per day | rs141623684 | 179113694 | A | C | -0.9562 | 0.2136 | 7.569E-06 | 20.039874 |
| Cigarettes smoked per day | rs17007092 | 124501466 | A | G | -0.5513 | 0.1175 | 2.72E-06 | 22.014065 |
| Cigarettes smoked per day | rs76917347 | 109926057 | A | G | -0.7955 | 0.1743 | 5.024E-06 | 20.829824 |
| Cigarettes smoked per day | rs2898605 | 106462217 | G | A | -0.4706 | 0.1025 | 4.392E-06 | 21.079297 |
| Cigarettes smoked per day | rs191235745 | 137832119 | T | A | -0.7471 | 0.1663 | 7.083E-06 | 20.182408 |
| Cigarettes smoked per day | rs2560227 | 152676677 | A | G | -0.4915 | 0.0894 | 3.89E-08 | 30.225397 |
| Cigarettes smoked per day | rs9387143 | 97622608 | A | G | -0.3738 | 0.0785 | 1.921E-06 | 22.674582 |
| Cigarettes smoked per day | rs529633143 | 75095783 | G | A | -1.2226 | 0.2519 | 1.207E-06 | 23.556591 |
| Cigarettes smoked per day | rs560315 | 168143639 | A | T | -0.477 | 0.1059 | 6.694E-06 | 20.288262 |
| Cigarettes smoked per day | rs211618 | 133726742 | T | A | -0.4255 | 0.0924 | 4.068E-06 | 21.205826 |
| Cigarettes smoked per day | rs35134443 | 126065 | C | G | 0.2985 | 0.0656 | 5.325E-06 | 20.705274 |
| Cigarettes smoked per day | rs4598257 | 19974049 | C | T | -0.7965 | 0.1495 | 9.991E-08 | 28.385018 |
| Cigarettes smoked per day | rs2898363 | 13395345 | G | C | 1.2749 | 0.2869 | 8.866E-06 | 19.746546 |
| Cigarettes smoked per day | rs16920732 | 57448712 | T | A | -0.3207 | 0.0699 | 4.425E-06 | 21.049586 |
| Cigarettes smoked per day | rs11139773 | 85393690 | T | C | -0.9089 | 0.1874 | 1.231E-06 | 23.523018 |
| Cigarettes smoked per day | rs10814479 | 36930247 | A | G | -0.3691 | 0.082 | 6.686E-06 | 20.260977 |
| Cigarettes smoked per day | rs522422 | 113633523 | T | C | -0.3293 | 0.0744 | 9.481E-06 | 19.590142 |
| Cigarettes smoked per day | rs12257716 | 35552072 | C | T | 0.3567 | 0.0807 | 9.841E-06 | 19.537057 |
| Cigarettes smoked per day | rs10509742 | 102090924 | T | A | -0.7191 | 0.1459 | 8.254E-07 | 24.292264 |
| Cigarettes smoked per day | rs56294630 | 73345718 | A | G | -0.5651 | 0.1141 | 7.247E-07 | 24.528971 |
| Cigarettes smoked per day | rs12146344 | 53400748 | G | T | 0.9786 | 0.2017 | 1.222E-06 | 23.539575 |
| Cigarettes smoked per day | rs34894498 | 7803147 | G | A | -0.9652 | 0.2086 | 3.704E-06 | 21.409475 |
| Cigarettes smoked per day | rs147831977 | 86887385 | T | C | -0.5033 | 0.1124 | 7.596E-06 | 20.050317 |
| Cigarettes smoked per day | rs61856676 | 33303335 | G | A | -0.5982 | 0.1336 | 7.559E-06 | 20.048408 |
| Cigarettes smoked per day | rs1520898 | 20236517 | C | T | 0.4274 | 0.0945 | 6.147E-06 | 20.45528 |
| Cigarettes smoked per day | rs78006379 | 2358899 | T | C | -1.2761 | 0.2846 | 7.31E-06 | 20.104794 |
| Cigarettes smoked per day | rs115809635 | 46602778 | A | T | -1.1405 | 0.2423 | 2.513E-06 | 22.155612 |
| Cigarettes smoked per day | rs4767557 | 117972643 | G | A | 0.3414 | 0.0715 | 1.805E-06 | 22.798955 |
| Cigarettes smoked per day | rs117016831 | 24552666 | G | T | -1.198 | 0.2385 | 5.095E-07 | 25.23114 |
| Cigarettes smoked per day | rs9546647 | 84880356 | C | T | -0.9858 | 0.2225 | 9.419E-06 | 19.629878 |
| Cigarettes smoked per day | rs9546710 | 85052568 | T | G | -0.4199 | 0.0916 | 4.545E-06 | 21.013616 |
| Cigarettes smoked per day | rs61744423 | 52923820 | C | T | -0.761 | 0.1723 | 9.981E-06 | 19.507372 |
| Cigarettes smoked per day | rs75316763 | 31810715 | T | C | -1.1107 | 0.2501 | 8.99E-06 | 19.722691 |
| Cigarettes smoked per day | rs72740955 | 78849779 | T | C | 0.7264 | 0.0669 | 1.903E-27 | 117.8961 |
| Cigarettes smoked per day | rs4786368 | 1648638 | C | G | -0.298 | 0.0668 | 8.094E-06 | 19.901216 |
| Cigarettes smoked per day | rs55807389 | 66794440 | A | G | -0.5575 | 0.1242 | 7.163E-06 | 20.148678 |
| Cigarettes smoked per day | rs56113850 | 41353107 | C | T | 0.3698 | 0.0657 | 1.822E-08 | 31.681306 |
| Cigarettes smoked per day | rs5753908 | 32669819 | C | T | -0.3174 | 0.0718 | 9.823E-06 | 19.541818 |

**Abbreviations:** Chr: chromosome; EA: effect Allele; EAF: effect allele frequency; OA: other Allele; SE: standard error.

**Supplementary Table S5** | Causal effect of Lung Cancer on major depression and schizophrenia.

| **Exposure** | **Outcome** | **Method** | **Number of IVs** | **OR (95% CI)** | **P-Value** |
| --- | --- | --- | --- | --- | --- |
| Overall lung cancer | Major depression | IVW | 4 | 0.959 (0.872, 1.055) | 0.393 |
|  |  | WM | 4 | 1.006 (0.968, 1.047) | 0.749 |
|  |  | MR Egger | 4 | 1.006 (0.722, 1.403) | 0.975 |
|  | Schizophrenia | IVW | 3 | 0.891 (0.544, 1.458) | 0.646 |
|  |  | WM | 3 | 1.054 (0.975, 1.139) | 0.185 |
|  |  | MR Egger | 3 | 1.308 (0.036, 47.155) | 0.907 |
| Lung adenocarcinoma | Major depression | IVW | 29 | 1.004 (0.991, 1.017) | 0.571 |
|  |  | WM | 29 | 0.995 (0.981, 1.010) | 0.548 |
|  |  | MR Egger | 29 | 1.000 (0.974, 1.027) | 0.995 |
|  | Schizophrenia | IVW | 22 | 1.025 (0.992, 1.058) | 0.142 |
|  |  | WM | 22 | 1.010 (0.975, 1.045) | 0.589 |
|  |  | MR Egger | 22 | 1.050 (0.975, 1.131) | 0.208 |
| Squamous cell lung cancer | Major depression | IVW | 23 | 0.985 (0.968, 1.002) | 0.083 |
|  |  | WM | 23 | 0.995 (0.978, 1.013) | 0.585 |
|  |  | MR Egger | 23 | 0.985 (0.946, 1.027) | 0.488 |
|  | Schizophrenia | IVW | 22 | 1.004 (0.933, 1.080) | 0.920 |
|  |  | WM | 22 | 1.021 (0.979, 1.066) | 0.332 |
|  |  | MR Egger | 22 | 1.017 (0.835, 1.238) | 0.868 |

**Abbreviations:** 95% CI: 95% confidence interval; IV: instrumental variables; IVW: inverse-variance weighted; OR: odds ratio; WM: weighted median.

**Supplementary Table S6** | Effects of major depression and schizophreniaon on lung cancer after regulating smoking-related behaviors by multivariate Mendelian randomization analysis.

| **Exposure** | **Outcome** | **Method** | **Number of IVs** | **OR (95% CI)** | **P-Value** |
| --- | --- | --- | --- | --- | --- |
| Major depression | Overall lung cancer | IVW | 34 | 0.923 (0.694, 1.227) | 0.581 |
|  | Lung adenocarcinoma | IVW | 34 | 0.975 (0.654, 1.455) | 0.903 |
|  | Squamous cell lung cancer | IVW | 34 | 1.216 (1.259, 1.174) | 0.928 |
| Schizophrenia | Overall lung cancer | IVW | 119 | 1.060 (0.989, 1.135) | 0.099 |
|  | Lung adenocarcinoma | IVW | 119 | 1.036 (0.944, 1.138) | 0.450 |
|  | Squamous cell lung cancer | IVW | 119 | 1.007 (0.917, 1.107) | 0.876 |

**Abbreviations:** 95% CI: 95% confidence interval; BMI: body mass index; IV: instrumental variables; IVW: inverse-variance weighted; OR: odds ratio.

**Supplementary Table S7** | Sensitivity analysis on the association between major depression, schizophrenia and Lung Cancer.

| **Exposure** | **Outcome** | **Method** | **P-Value** |
| --- | --- | --- | --- |
| Major depression | Overall lung cancer | Heterogeneity Test | 0.677 |
|  |  | Pleiotropy Test | 0.060 |
|  |  | MR-PRESSO Test | 0.439 |
|  | Lung adenocarcinoma | Heterogeneity Test | 0.173 |
|  |  | Pleiotropy Test | 0.683 |
|  |  | MR-PRESSO Test | 0.162 |
|  | Squamous cell lung cancer | Heterogeneity Test | 0.214 |
|  |  | Pleiotropy Test | 0.151 |
|  |  | MR-PRESSO Test | 0.194 |
| Schizophrenia | Overall lung cancer | Heterogeneity Test | 0.282 |
|  |  | Pleiotropy Test | 0.832 |
|  |  | MR-PRESSO Test | 0.152 |
|  | Lung adenocarcinoma | Heterogeneity Test | 0.165 |
|  |  | Pleiotropy Test | 0.730 |
|  |  | MR-PRESSO Test | 0.073 |
|  | Squamous cell lung cancer | Heterogeneity Test | 0.443 |
|  |  | Pleiotropy Test | 0.684 |
|  |  | MR-PRESSO Test | 0.454 |

**Abbreviations:** MR-PRESSO: MR Pleiotropy RESidual Sum and Outlier.

**Supplementary Table S8** | Sensitivity analysis on the association between Lung Cancer and major depression/schizophrenia.

| **Exposure** | **Outcome** | **Method** | **P-Value** |
| --- | --- | --- | --- |
| Overall lung cancer | Major depression | Heterogeneity Test | 0.731 |
|  |  | Pleiotropy Test | 0.793 |
|  |  | MR-PRESSO Test | 0.311 |
|  | Schizophrenia | Heterogeneity Test | 0.937 |
|  |  | Pleiotropy Test | 0.866 |
|  |  | MR-PRESSO Test | NA |
| Lung adenocarcinoma | Major depression | Heterogeneity Test | 0.517 |
|  |  | Pleiotropy Test | 0.750 |
|  |  | MR-PRESSO Test | 0.381 |
|  | Schizophrenia | Heterogeneity Test | 0.509 |
|  |  | Pleiotropy Test | 0.472 |
|  |  | MR-PRESSO Test | 0.464 |
| Squamous cell lung cancer | Major depression | Heterogeneity Test | 0.151 |
|  |  | Pleiotropy Test | 0.975 |
|  |  | MR-PRESSO Test | 0.965 |
|  | Schizophrenia | Heterogeneity Test | 0.322 |
|  |  | Pleiotropy Test | 0.889 |
|  |  | MR-PRESSO Test | 0.952 |

**Abbreviations:** MR-PRESSO: MR Pleiotropy RESidual Sum and Outlier.
